# Association between ocular surface microbiota species and type 2 diabetes mellitus with or without retinopathy and related clinical parameters

**DOI:** 10.1128/msphere.00003-26

**Published:** 2026-05-12

**Authors:** Jihong Wang, Na Zhang, Jun-Jian Li, Yun-Yun Jin, Shi Huang, Jian-Huan Chen

**Affiliations:** 1 Department of Ophthalmology, Affiliated Hospital of Jiangnan University, Wuxi, China; 2 Laboratory of Genomic and Precision Medicine, Wuxi School of Medicine, Jiangnan University 66374, Wuxi, Jiangsu, China; 3 Joint Primate Research Center for Chronic Diseases, Institute of Zoology of Guangdong Academy of Science, Jiangnan University 66374, Wuxi, Jiangsu, China; 4 MOE Medical Basic Research Innovation Center for Gut Microbiota and Chronic Diseases, Wuxi School of Medicine, Jiangnan University 66374, Wuxi, Jiangsu, China; 5 Jiangnan University-Xinshijie Eye Hospital Joint Ophthalmic Research Center, Wuxi, China; 6 Faculty of Dentistry, The University of Hong Kong, Hong Kong, China; University of Michigan-Ann Arbor, Ann Arbor, Michigan, USA

**Keywords:** type 2 diabetes mellitus, diabetic retinopathy, ocular surface microbiota, species-resolved profiling, fungi

## Abstract

Diabetic retinopathy (DR) is the most prevalent blinding microvascular complication of diabetes mellitus. Although altered ocular surface microbiota (OSM) has been recently linked to type 2 diabetes mellitus (DM) and diabetic retinopathy (DR), its involvement in these diseases remains to be elucidated at the species level. Our current study investigated 92 conjunctival sac swabs from 28 nondiabetic controls (NC) and 64 type 2 DM patients, including 31 with DR and 33 without DR (non-DR, NDR), for species-resolved OSM profiles using 2bRAD-M sequencing, and investigated their correlations with clinical parameters. A total of 232 species were identified in the study, with 12.5% and 10.8% exclusively observed in DR and NDR, respectively. Significant differences in beta diversity, but not in alpha diversity, were observed in DR compared to NDR and NC. Substantial changes in OSM species composition were observed in DM compared to NC, most attributable to DR. Commensal bacterial species, especially Actinomycetota species, such as *Bifidobacterium infantis,* and fungal species, such as *Malassezia restricta,* substantially decreased in DM. In contrast, species mainly belonging to the Pseudomonadota phylum, such as *Pseudomonas* and *Pseudomonas aeruginosa,* increased in DM, especially in DR. Moreover, such differential OSM species exhibited a significant correlation with clinical parameters, especially those associated with blood sugar indices, kidney complications, or insulin treatment, with *Acinetobacter johnsonii* being the most positively correlated with glycosylated hemoglobin A1c. Our current study revealed substantially altered OSM species profiles associated with DM/DR, suggesting a potential role for OSM species and providing new insights into the clinical correlation and diagnostic value of OSM.

**IMPORTANCE:**

Our research characterizes the ocular surface microbiota (OSM) at the species level in patients with type 2 diabetes mellitus (DM), both with and without diabetic retinopathy (DR and NDR), using 2bRAD-M sequencing. It uncovers unique microbial signatures, notably the reduction of commensal Actinomycetota and *Malassezia restricta*, and the increase of opportunistic Pseudomonadota such as *Pseudomonas aeruginosa* and *Acinetobacter johnsonii*. These microbial changes are linked to glycemic control, kidney function, and systemic inflammation, connecting local ocular microbiota to systemic microvascular damage. The study also highlights OSM species with strong diagnostic potential for DM and DR, indicating that noninvasive, microbiota-based biomarkers could supplement existing imaging-based screening. Overall, these findings deepen the understanding of host-microbiota interactions in diabetes and retinopathy and support future research into mechanisms, longitudinal effects, and microbiota-targeted therapies to prevent or reduce vision-threatening diabetic complications.

## INTRODUCTION

Type 2 diabetes mellitus (DM) is a chronic metabolic disorder with persistent hyperglycemia resulting from either relative or absolute insulin deficiency (1). Sustained hyperglycemia can lead to damage in various organ systems over time, resulting in diabetic complications. Diabetic retinopathy (DR) is a common blinding microvascular complication of DM in the elderly and working-age individuals, resulting from damage to the retinal blood vessels caused by prolonged hyperglycemia exposure (2). DR was estimated to affect 22.27% of the global diabetic population in 2020 (3) and 16.3% of adult diabetic patients in 2022 in China (4), with a significant impact on public health. Multiple predisposing factors could be involved in DR, including the course of DM (5), glycosylated hemoglobin (6), hypertension (7), chronic kidney disease (8), and dyslipidemia, the study of which remains challenging for the prevention and treatment of DR. Additionally, current DR diagnosis relies on costly fundus photography and examination, which require specialized equipment and expertise.

Ocular surface microbiota (OSM) refers to the microbial community residing on the ocular surface, which is constantly exposed to various microorganisms, allergens, and other environmental factors (9). The OSM mainly comprises Firmicutes, Actinomycetota, Bacteroidetes, and Pseudomonadota, which play a crucial role in maintaining the ocular surface’s homeostasis and health (10–12). Emerging evidence indicates that OSM dysbiosis is involved in DM and various ocular surface diseases, including dry eye disease, meibomian gland dysfunction, allergic conjunctivitis, and DR (10, 13–18). Zhang et al. found differences in the tear microbiota between DM and DM with dry eye disease (19). However, these OSM studies in DM and DR are based on the bacterial culture or 16S rRNA gene amplicon sequencing. Only a limited number of bacteria are culturable; 16S rRNA sequencing is used only for bacterial and archaeal analysis and has limited resolution at the species level. Additionally, the low biomass of OSM samples poses a challenge for metagenomic sequencing, which typically requires a high quantity and quality of environmental samples. Therefore, species-resolved OSM profiles remain essentially uninvestigated.

**Fig 1:**
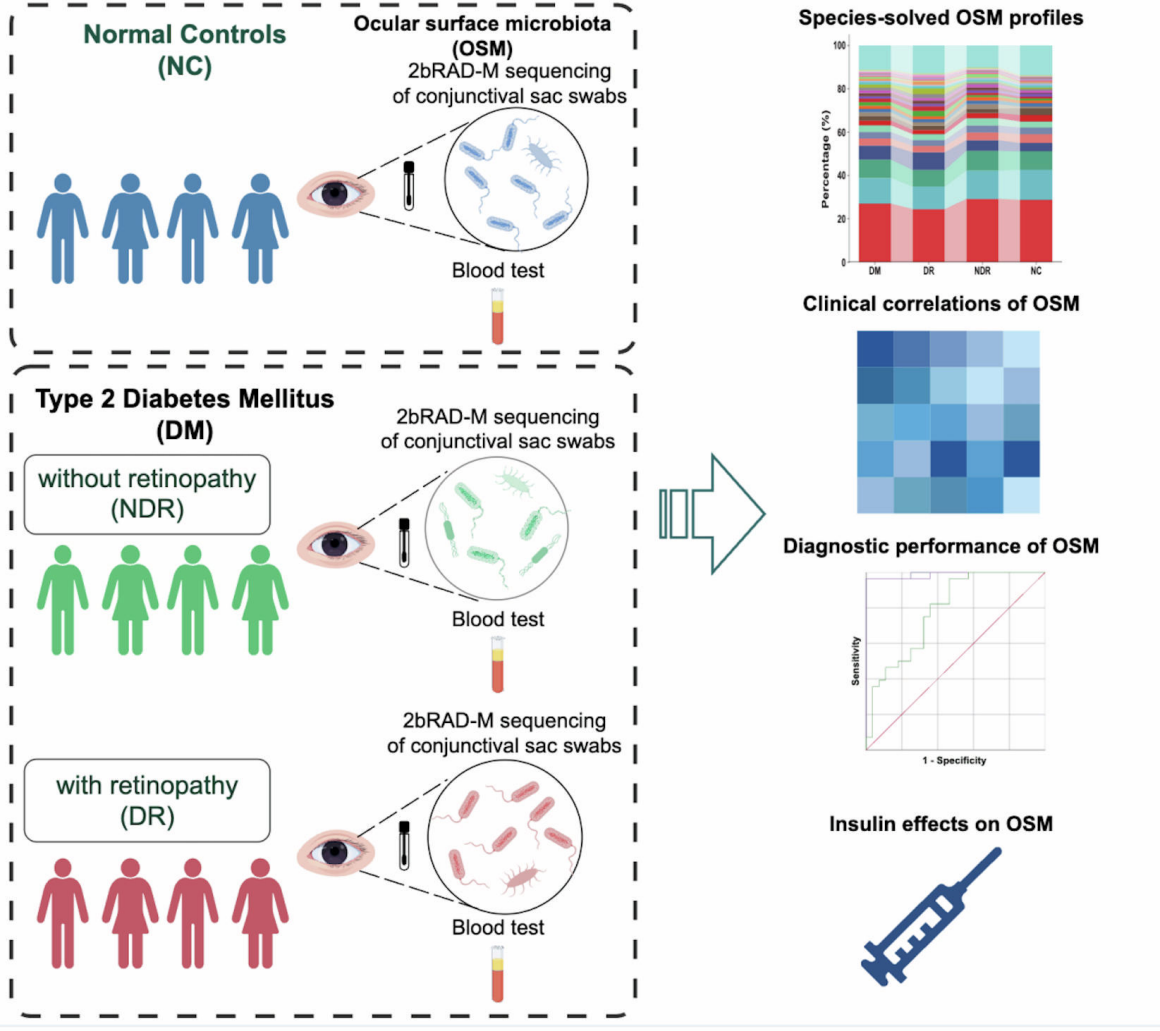
Overview of the study. The study profiles OSM at the species level, revealing significant compositional differences in type 2 DM with or without DR. Key disease-associated species correlate with clinical parameters, suggesting the potential for OSM profiling as a diagnostic tool for diabetes-related conditions.

2bRAD-M sequencing performs qualitative and relative quantitative analyses of unique tags obtained from the digestion of microbial genomes with type IIB restriction enzymes (20, 21), allowing for high resolution and sensitivity in microbial detection with satisfying species-level resolution (22, 23). In the current study, 2bRAD-M was used to investigate the OSM of conjunctival sac swabs from a cohort comprising nondiabetic controls (NC) and type 2 DM patients with or without retinopathy (DR or NDR) (Fig. 1). Our findings revealed species-resolved OSM profiles associated with DM or DR, as well as their correlations with blood test parameters, providing new insights into the OSM species-based noninvasive diagnosis of these disease conditions.

## MATERIALS AND METHODS

### Study subjects and sample collection

A cohort of 92 age- and gender-matched subjects was recruited, including 28 NC and 64 type 2 DM patients, composed of 31 DR and 33 NDR patients. All of the DM patients were diagnosed according to at least one of the following criteria: (i) fasting blood glucose ≥7.0 mmol/L (ii); 2-h blood glucose level in the oral glucose tolerance test (OGTT) ≥11.1 mmol/L (iii); random blood glucose ≥11.1 mmol/L; and (iv) glycosylated hemoglobin (HbA1c) ≥6.5% (24). All recruited subjects underwent examination using a noncontact ophthalmoscope with mydriasis, fundus photography, and optical coherence tomography (OCT). DM patients were diagnosed with DR if they had microaneurysms, retinal hemorrhage, or macular edema. Exclusion criteria for study subjects included antibiotic**/**steroid treatment within the past month, a history of autoimmune diseases, active ocular surface diseases such as conjunctivitis and dry eye disease, and other ocular diseases, except DR and senile cataracts.

All information and samples were collected from the participants with their written consent. Demographic data, medical history, and body mass index (BMI) (calculated as body weight divided by height squared) were obtained from all study subjects. All samples were collected at the Affiliated Hospital of Jiangnan University. Fasting peripheral blood samples were collected. Conjunctival sac swab samples were collected from the inferior fornix conjunctiva with disposable sterile swabs, avoiding touching the eyelashes and eyelid margins (14). Following collection, all swab samples were immediately placed in sterile tubes and kept on ice. They were transferred to a −80°C freezer within 1 h to prevent potential microbial shifts or DNA degradation. The samples were stored at −80°C for approximately 3 months before DNA extraction. All extractions were carried out in a consolidated timeframe to ensure consistency in sample handling.

### Blood tests

Parameters of the peripheral blood samples were measured on a Dxi800 automatic biochemical analyzer (Beckman): glycosylated hemoglobin A1c (HbA1c), fasting blood glucose (FBG), retinol-binding protein (RBP), cystatin C (Cys-C), uric acid (UA), estimated glomerular filtration rate (eGFR), urea, creatinine (CREA), β2-microglobulin (β2-MG), direct bilirubin (DBIL), indirect bilirubin (IBIL), total bilirubin (TBIL), neutrophil-to-lymphocyte ratio (NLR), platelet-to-lymphocyte ratio (PLR), hemoglobin (HBG), albumin (ALB), red blood cell (RBC), and hematocrit (HCT) were included.

### DNA extraction, 2bRAD-M library construction, sequencing, and processing

DNA was extracted from conjunctival sac swabs using a TIANamp Micro DNA Kit (Tiangen, China). The DNA purity and concentration were assessed by a NanoVue Plus spectrophotometer (GE Healthcare, China). 2bRAD-M libraries were constructed using the service provided by OE Bio (Qingdao, China), following a protocol modified from that reported by Wang et al. (21). In brief, DNA was digested with 4 U of BcgI (NEB) at 37°C for 3 h. The digested DNA was then ligated to adapters using the T4 DNA ligation master mix (NEB) at 4°C for 12 h, amplified by PCR, followed by purification with the QIAquick PCR purification kit (Qiagen) and sequencing on the NovaSeq 6000 (Illumina) platform with a configuration of paired-end 150-bp reads. The adapters and primers used for 2bRAD-M library preparation are summarized in Table S1. The generated sequencing reads were analyzed for species-specific 2bRAD markers using a pre-built comprehensive 2bRAD marker database, and relative taxonomic abundance was calculated as previously described (25). To control contamination during sequencing, our samples were handled in a strict, positive-pressure lab to prevent airflow contamination. Each step was performed in a dedicated room, with unidirectional sample transfer through wall windows. Negative environmental controls at multiple stages were processed with samples to help identify background contamination. Self-developed decontamination software ensured data reliability.

### Statistical analysis

SPSS 25.0 and GraphPad Prism software were used for statistical analysis. The chi-square test and the Kruskal-Wallis (KW) test were used to analyze the count data and continuous data, respectively. The Chao1, Shannon, and Simpson indices among the three groups were compared using One-way ANOVA. Beta diversity among groups was compared using permutational multivariate analysis of variance (PERMANOVA). Differential taxa among groups were analyzed using the KW test and the linear discriminant analysis effect size (LEfSe) algorithm with a LDA score cutoff >2.0. Correlations between clinical characteristics and OSM taxa abundances were analyzed using the Spearman correlation method. A cutoff of *P* < 0.05 was applied for statistical significance.

## RESULTS

### Demographic and blood test parameters of the study subjects

The demographic and blood test parameters of the study subjects are summarized in Table 1. All three groups had an average age over 60 years, with no significant differences in gender, age, or BMI (KW test, *P* > 0.05). Significant differences were found in FBG, HbA1c, Cys-C, UA, β2-MG, urea, CREA, RBP, DBIL, IBIL, TBIL, NLR, ALB, and HCT among the three groups (KW test*, P* < 0.05). Significant differences were found between DM and NC in FBG, HbA1c, β2-MG, DBIL, and TBIL. Cys-C, UA, urea, β2-MG, RBP, IBIL, NLR, and PLR were significantly higher, whereas eGFR, DBIL, and ALB were lower in DR than NDR (*P* < 0.05). Notably, Cys-C showed significant opposite changes in DR and NDR compared to NC, with the highest and lowest levels found in DR and NDR, respectively (*P* < 0.05).

**TABLE 1:** Demographic and blood test parameters of the study subjects^*a*^

Parameter	DM (*n* = 64)	DR (*n* = 31)	NDR (*n* = 33)	NC (*n* = 28)	*P* (three-group comparison)	*P* (DM vs. NC)	*P* (DR vs. NDR)	*P* (DR vs. NC)	*P* (NDR vs. NC)
Gender, *n* (%)					0.221	0.871	0.084	0.486	0.323
Male	24 (37.50)	15 (48.39)	9 (27.27)	11 (39.29)	–	–	–	–	–
Female	40 (62.50)	16 (51.61)	24 (72.73)	17 (60.71)	–	–	–	–	–
Age, year	64.84 ± 8.11	63.16 ± 6.82	66.42 ± 8.77	66.61 ± 8.58	0.160	0.339	0.209	0.074	0.942
History of DM, year	–	10.74 ± 6.76	10.86 ± 7.21	–	–	–	0.859	–	–
BMI, kg/m^2^	24.46 ± 3.44	24.06 ± 2.84	24.83 ± 3.92	24.05 ± 3.52	0.570	0.684	0.515	1.00	0.487
FBG, mmol/L	7.63 ± 2.50	7.59 ± 2.17	7.67 ± 2.80	5.36 ± 1.14	**<0.001**	**<0.001**	0.573	**<0.001**	**<0.001**
HbA1c, %	8.32 ± 1.95	7.87 ± 1.60	8.74 ± 2.16	5.35 ± 7.13	**<0.001**	**<0.001**	0.204	**<0.001**	**<0.001**
Cys-C, mg/L	1.19 ± 0.98	1.59 ± 1.27	0.82 ± 0.29	1.02 ± 0.82	**<0.001**	0.581	**<0.001**	**0.004**	**<0.001**
UA, umol/L	340.37 ± 83.21	381.6 ± 66.24	301.57 ± 79.46	328.28 ± 64.07	**<0.001**	0.440	**<0.001**	**0.002**	0.099
eGFR, ml/min	101.64 ± 45.13	88.06 ± 50.33	114.40 ± 35.89	98.89 ± 25.45	0.065	0.611	0.039	0.354	0.080
Urea, mmol/L	7.67 ± 4.81	9.75 ± 6.07	5.71 ± 1.71	5.87 ± 1.17	**<0.001**	0.191	**<0.001**	**0.001**	0.329
CREA, umol/L	88.24 ± 66.73	113.07 ± 84.05	64.92 ± 18.46	72.26 ± 19.82	**0.023**	0.905	**0.008**	0.182	0.140
β2-MG, mg/L	3.45 ± 2.72	4.33 ± 3.47	2.62 ± 1.36	2.20 ± 0.51	**0.006**	**0.016**	0.027	**0.003**	0.208
RBP, mg/L	44.66 ± 13.20	49.10 ± 14.91	40.49 ± 9.87	43.06 ± 12.08	**0.012**	0.084	**0.016**	**0.007**	0.717
DBIL, µmol/L	2.93 ± 2.75	2.10 ± 1.09	3.70 ± 3.54	2.59 ± 0.82	**<0.001**	0.709	**<0.001**	**0.008**	0.060
IBIL, µmol/L	7.44 ± 4.18	8.73 ± 3.84	6.23 ± 4.18	4.18 ± 11.55	**<0.001**	**<0.001**	**0.006**	**0.011**	**<0.001**
TBIL, µmol/L	9.85 ± 4.66	10.41 ± 4.80	9.32 ± 4.54	14.10 ± 4.71	**<0.001**	**<0.001**	0.262	**0.004**	**<0.001**
NLR	2.50 ± 2.16	2.65 ± 0.99	2.36 ± 2.87	2.06 ± 1.26	**0.001**	0.277	**<0.001**	**0.006**	0.456
PLR	120.78 ± 57.57	131.19 ± 56.22	111.01 ± 57.95	114.85 ± 37.57	0.121	0.608	0.040	0.643	0.188
HBG, g/L	124.31 ± 21.23	120.1 ± 23.30	128.18 ± 18.62	133.43 ± 15.03	0.059	0.050	0.134	0.028	0.213
ALB, g/L	38.27 ± 5.59	36.98 ± 4.74	39.48 ± 6.10	39.35 ± 3.94	**0.034**	0.115	0.045	**0.014**	0.783
RBC, 10^−12^/L	4.16 ± 0.72	3.98 ± 0.73	4.34 ± 0.66	4.25 ± 0.51	0.100	0.647	0.047	0.122	0.487
HCT, %	37.26 ± 6.23	35.78 ± 6.77	38.55 ± 5.43	39.55 ± 4.34	**0.040**	0.108	0.037	0.027	0.529
Schirmer I, mm	14.17 ± 1.27	14.28 ± 1.29	14.06 ± 1.25	14.43 ± 1.21	0.802	0.428	0.658	0.559	0.523
TBUT, s	15.36 ± 1.68	15.19 ± 1.73	15.53 ± 1.63	15.54 ± 1.07	0.642	0.840	0.422	0.465	0.985

^*a*^ *P*-values are calculated from the KW test. Boldface indicates significant *P*-values for overall significance or after *post-hoc* Bonferroni adjustments for pairwise comparisons. –, not applicable; BMI, body mass index; HbA1c, glycosylated hemoglobin; FBG, fasting blood glucose; RBP, retinol-binding protein; Cys-C, cystatin c; UA, uric acid; eGFR, estimated glomerular filtration rate; CREA, creatinine; β2-MG, β2-microglobulin; DBIL, direct bilirubin; IBIL, indirect bilirubin; TBIL, total bilirubin; NLR, neutrophil-to-lymphocyte ratio; HBG, hemoglobin; ALB, albumin; PLR, platelet-to-lymphocyte ratio; RBC, red blood cell count; HCT, hematocrit ; TBUT, tear break-up time.

### OSM diversities in the DM/DR cohort

**Fig 2:**
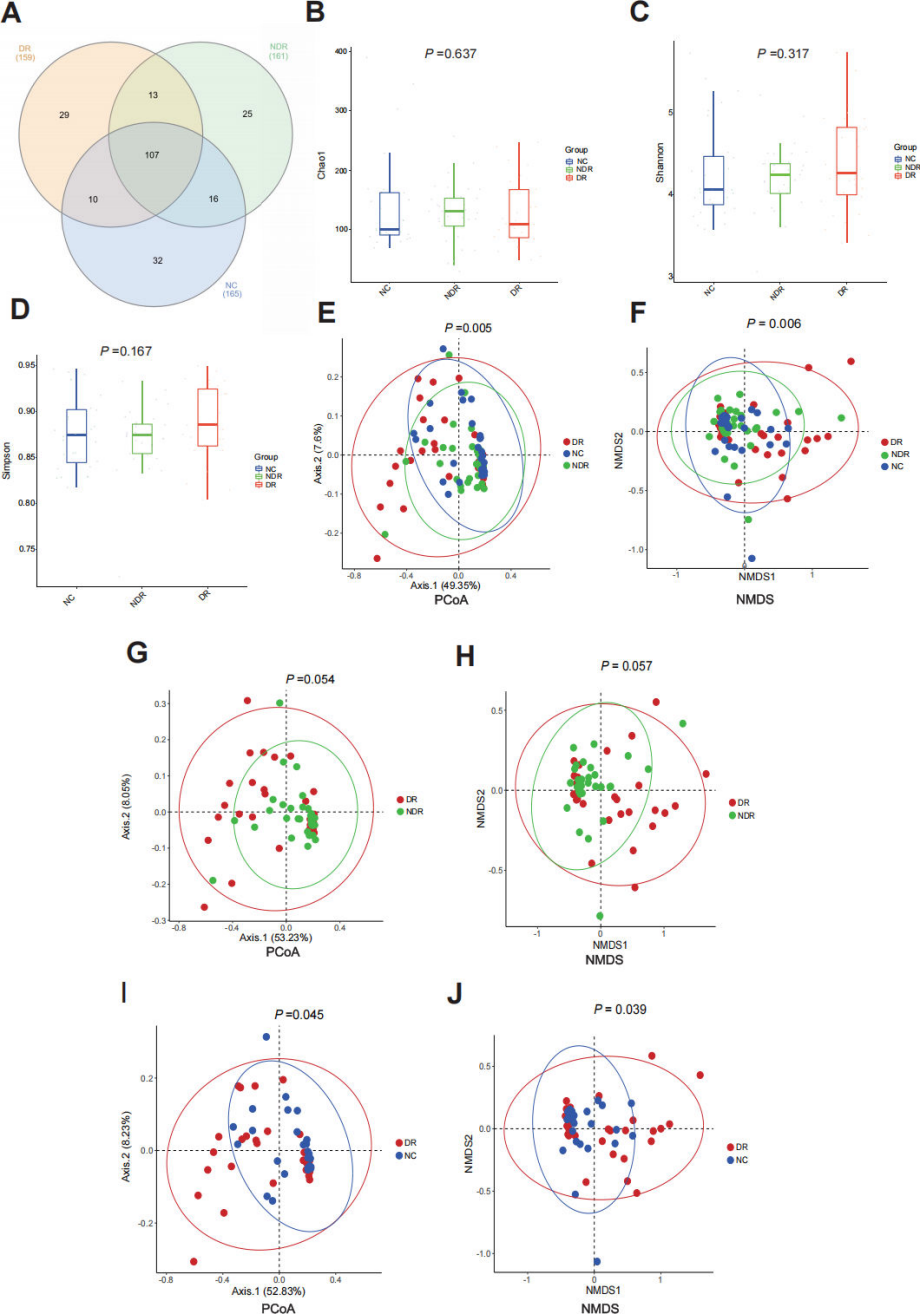
Alpha and beta diversities of the OSM in NC (*n* = 28), NDR (*n* = 33), and DR (*n* = 31). (**A**) Venn diagram showing the comparison of species among the three groups. Alpha diversity metrics, including (**B**) the Chao1, (**C**) Shannon, and (**D**) Simpson indices, were compared among the three groups. (**E and F**) Bray-Curtis distance-based PCoA and NMDS analysis of the three groups, (**G and H**) pairwise comparisons between DR and NC groups, and (**I and J**) between DR and NDR groups. The pairwise *P*-values are corrected using the Bonferroni method.

Table S2 summarizes the 2bRAD-M sequencing data of our study cohort. A total of 232 species were detected from the conjunctival sac swabs based on the criteria of average relative abundance >0.0001 and detection of the species in at least 5 samples per group. The Venn diagram in Fig. 2A shows that 107 species are shared across DR, NDR, and NC, with 29, 25, and 32 species exclusively found in DR, NDR, and NC, respectively. Alpha diversity analysis of the OSM found no significant differences in the Chao1, Shannon, or Simpson indices among the three groups (Fig. 2B through D). Nevertheless, principal coordinate analysis (PCoA) and nonmetric multidimensional scaling (NMDS) based on Bray-Curtis distances revealed significant differences in beta diversity among the three groups (Fig. 2E through F), with substantial pair-wise differences between DR and NC (*P* < 0.05) and marginal significant difference between DR and NDR (*P* < 0.06) (Fig. 2G through J), but not between NC and NDR (*P* > 0.45, data not shown).

### Taxonomic composition of the OSM in the DM/DR cohort

**Fig 3:**
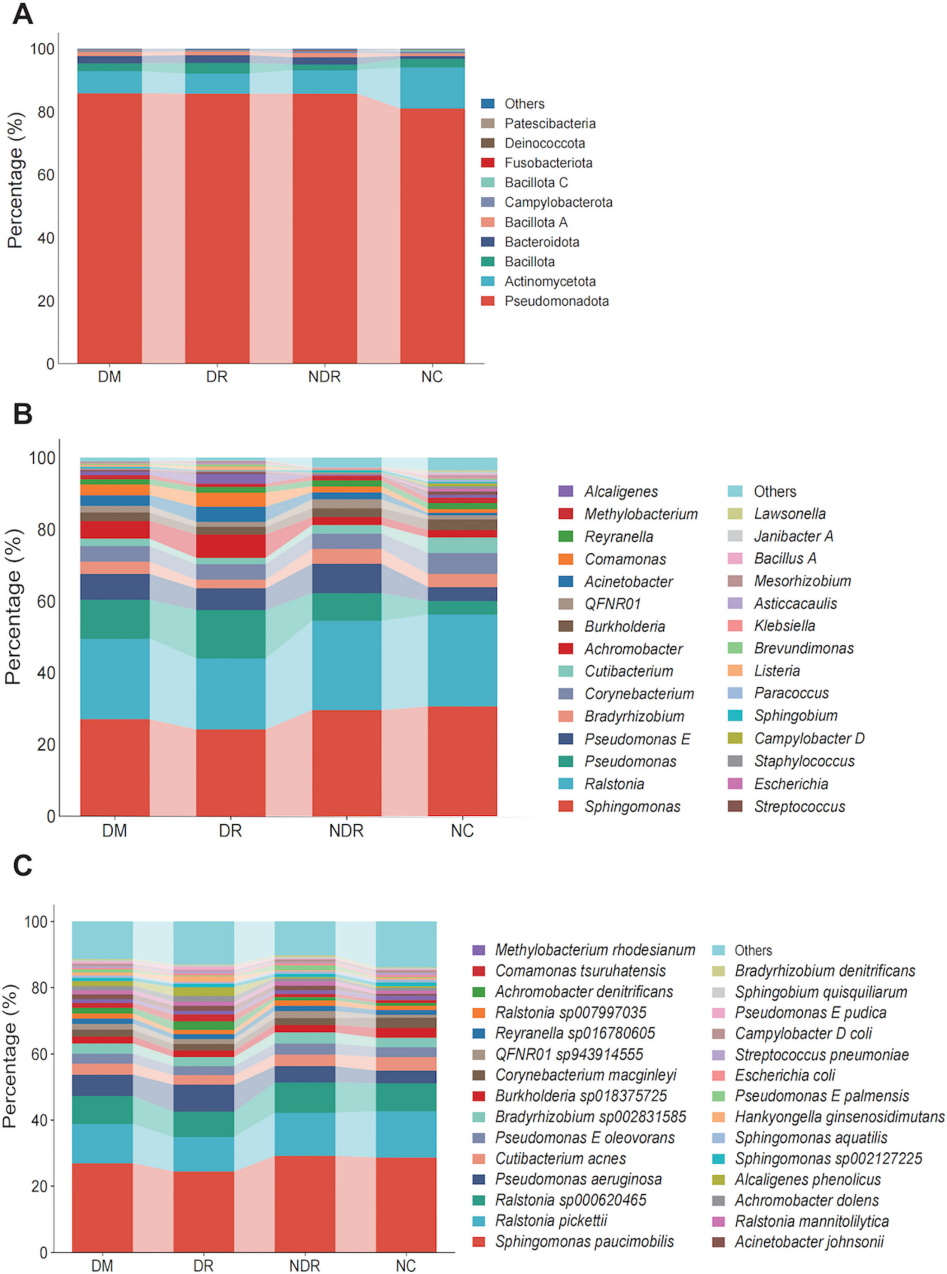
Taxonomic composition of the OSM in DM (*n* = 64), DR (*n* = 31), NDR (*n* = 33), and NC (*n* = 28). The relative abundance is shown at the (**A**) phylum, (**B**) genus, and (**C**) species levels.

For bacteria and archaea in the OSM, the two top phyla were Pseudomonadota (85.92%, 85.79%, 85.80%, and 81.09% in DM, DR, NDR, and NC, respectively) and Actinomycetota (6.94%, 6.36%, 7.48%, and 12.98% in DM, DR, NDR, and NC, respectively) (Fig. 3A). The top two dominant genera were *Sphingomonas* (27.02%, 24.16%, 29.56%, and 30.60% in DM, DR, NDR, and NC, respectively) and *Ralstonia* (22.52%, 19.82%, 24.93%, and 25.65% in DM, DR, NDR, and NC, respectively) (Fig. 3B). At the species level, *Sphingomonas paucimobilis*, *Ralstonia pickettii*, *Ralstonia sp00062046*, *Pseudomonas aeruginosa,* and *Cutibacterium acnes* were the top five abundant bacterial species (Fig. 3C).

For fungi in the OSM, Ascomycota and Basidiomycota were the top two dominant phyla across all groups. *Malassezia* and *Malassezia restricta* were the most predominant genus and species across all groups, respectively (Fig. S1 and S2).

### Differential OSM taxa in the DM cohort

**Fig 4:**
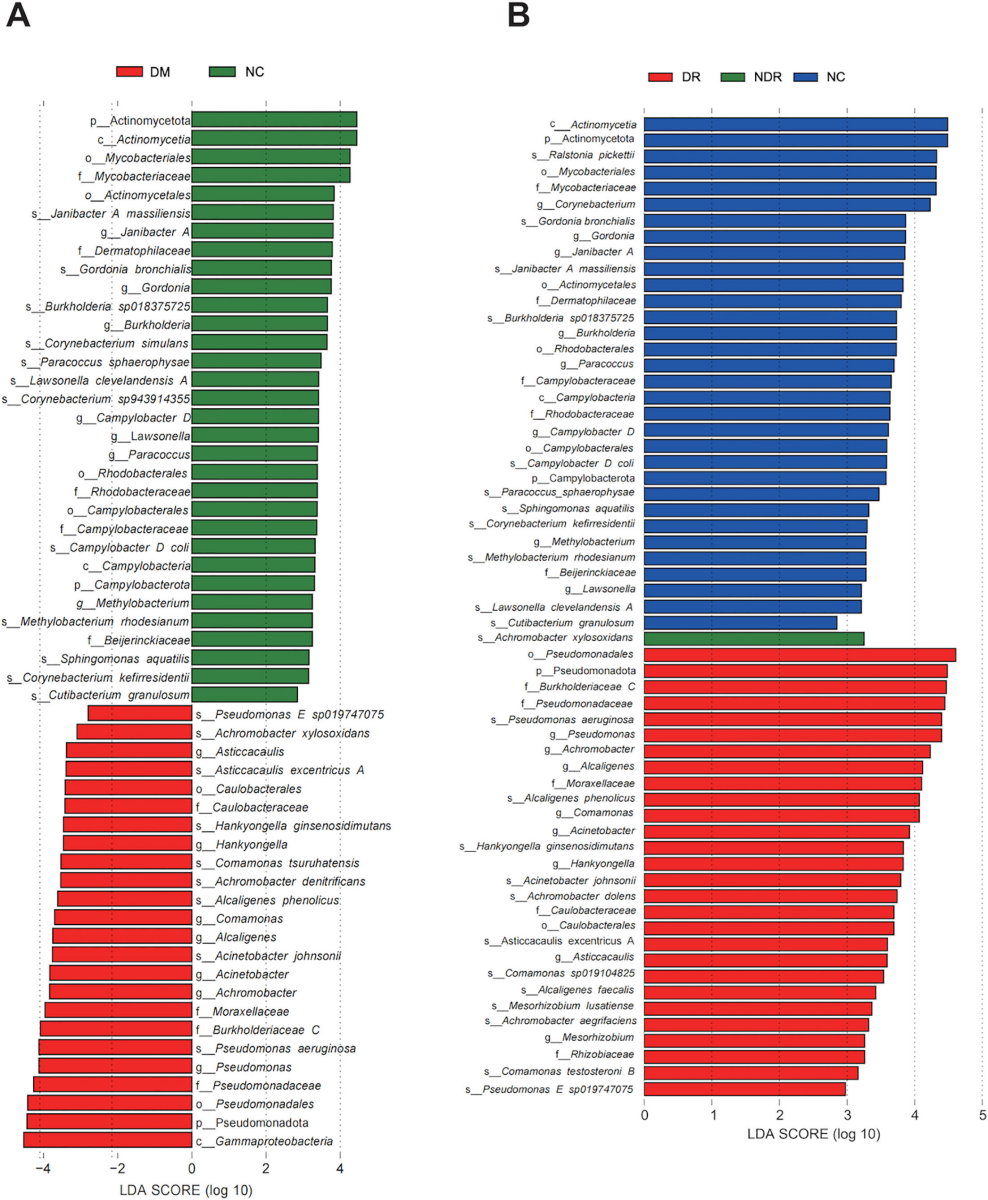
Differentially enriched taxa in groups identified by the LEfSe algorithm. Differential taxa with LDA > 3.0 are shown (**A**) for the comparison between DM and NC and (**B**) among DR, NDR, and NC. The complete lists of differential taxa are shown in Fig. S3.

The LEfSe algorithm was then employed to explore the differential OSM among groups further (Fig. 4). First, OSM taxa were compared between DM and NC (Fig. 4A). No differential archaeal taxa were observed between DM and NC. Bacteria taxa enriched in NC included 3 phyla: Actinomycetota, Campylobacterota, and Bacillota C; 10 families such as Mycobacteriaceae, Dermatophilaceae, and Rhodobacteraceae; 19 genera, such as *Burkholderia, Janibacter A,* and *Gordonia*; and 39 bacterial species, such as *Burkholderia sp018375725, Janibacter A massiliensis,* and *Gordonia bronchiali*, which were enriched in NC. Notably, the fungal species *Malassezia restricta* and the genus *Malassezia* (family Malasseziaceae) and Basidiomycota were exclusively enriched in NC.

In contrast, taxa enriched in DM comprised one phylum, Pseudomonadota; six bacterial families, such as Pseudomonadaceae, Burkholderiaceae, C, and Moraxellaceae; eleven genera, such as *Pseudomonas, Achromobacter,* and *Acinetobacter*; and sixteen species, such as *Pseudomonas aeruginosa, Acinetobacter johnsonii,* and *Alcaligenes phenolicus* (Fig. 4A).

LEfSe analysis was also conducted by dividing DM patients into NDR and DR to explore the association between OSM and retinopathy (Fig. 4B). Notably, more taxa were enriched in DR than in NDR. Among the three groups, taxa enriched in DR included two phyla, including Pseudomonadota and Bacillota A; eight families, such as Burkholderiaceae C, Pseudomonadaceae, and Moraxellaceae; eighteen genera, such as *Pseudomonas, Achromobacter,* and *Alcaligenes*; and twenty-four species, such as *Pseudomonas aeruginosa, Alcaligenes phenolicus,* and *Hankyongella ginsenosidimutans*. In contrast, only seven species, such as *Cupriavidus sp018729255, Achromobacter xylosoxidans,* and *Reyranella sp900110395*, but not other taxa, were enriched in NDR.

### Diagnostic performance of DM-associated OSM species

**Fig 5:**
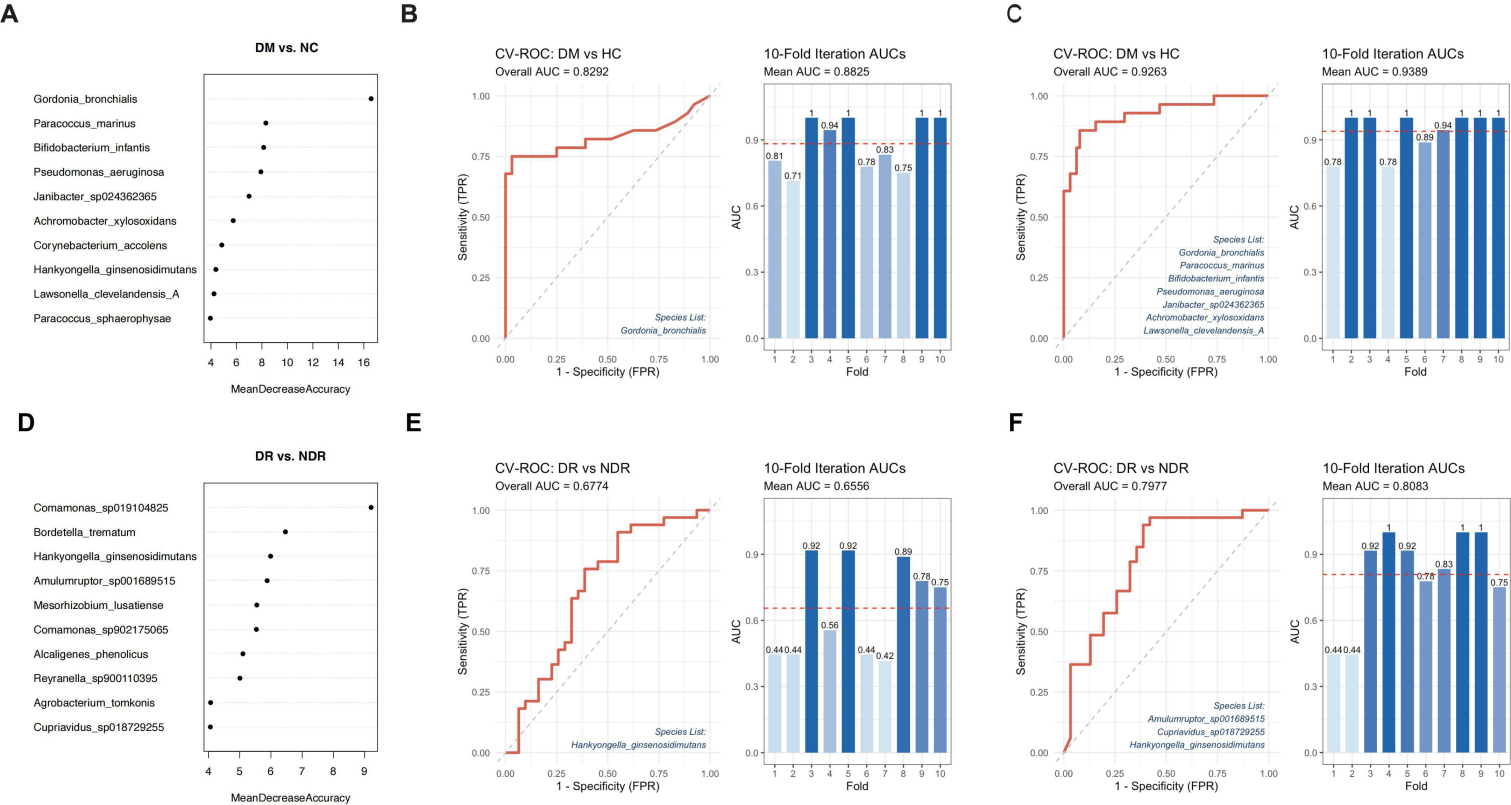
Prioritizing the differential OSM in distinguishing groups using random forest analysis and evaluation of their diagnosis performance. The top 10 differential OSM species in distinguishing DM from NC using random forest analysis (**A**). The ROCs of the top one and the best combination of species using 10-fold cross-validation in distinguishing DM from NC (**B and C**). The top 10 differential OSM species in distinguishing DR from NDR using random forest analysis (**D**). The ROCs of the top one and the best combination of species using 10-fold cross-validation in distinguishing DR from NDR (**E and F**).

Random forest (RF) analysis was then used to prioritize the differential species identified by LEfSe for distinguishing the groups**.** The top 10 differential species between DM and NC selected by RF are shown in Fig. 5A. Receiver operating characteristic (ROC) curves with 10-fold cross-validation were used to evaluate the diagnostic performance of the selected marker species. A classifier based on the top species, *Gordonia bronchialis*, reached an overall area under the curve (AUC) of 0.83 (Fig. 5B), and the best classifier based on *Gordonia bronchialis* and six other species together attained an overall AUC of 0.93 in distinguishing DM and NC (Fig. 5C).

Likewise, the top 10 differential species between DR and NDR selected by RF analysis are shown in Fig. 5D. A classifier based on the species *Hankyongella ginsenosidimutans* reached an overall AUC of 0.68 (Fig. 5E), and the best classifier based on 7 species together achieved an overall AUC of 0.83 in distinguishing DR and NDR (Fig. 5F).

### Association between differential OSM and clinical and demographic parameters in the DM cohort

**Fig 6:**
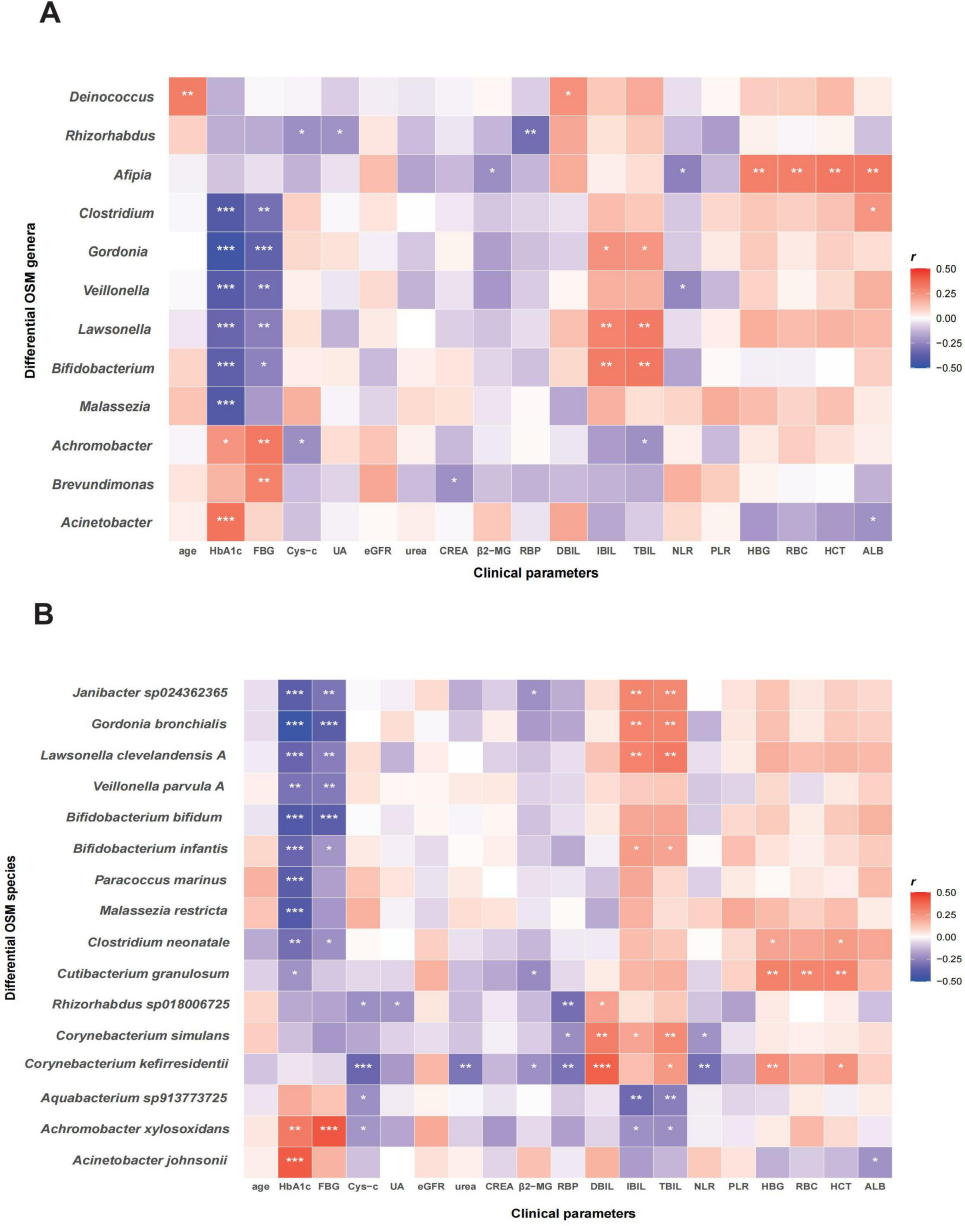
Association between the OSM and clinical parameters. Heatmaps showing Spearman correlations of differential (**A**) genera and (**B**) species with clinical parameters. *P < 0.05, ** *P* < 0.01, and *** *P* < 0.001.

**Fig 7:**
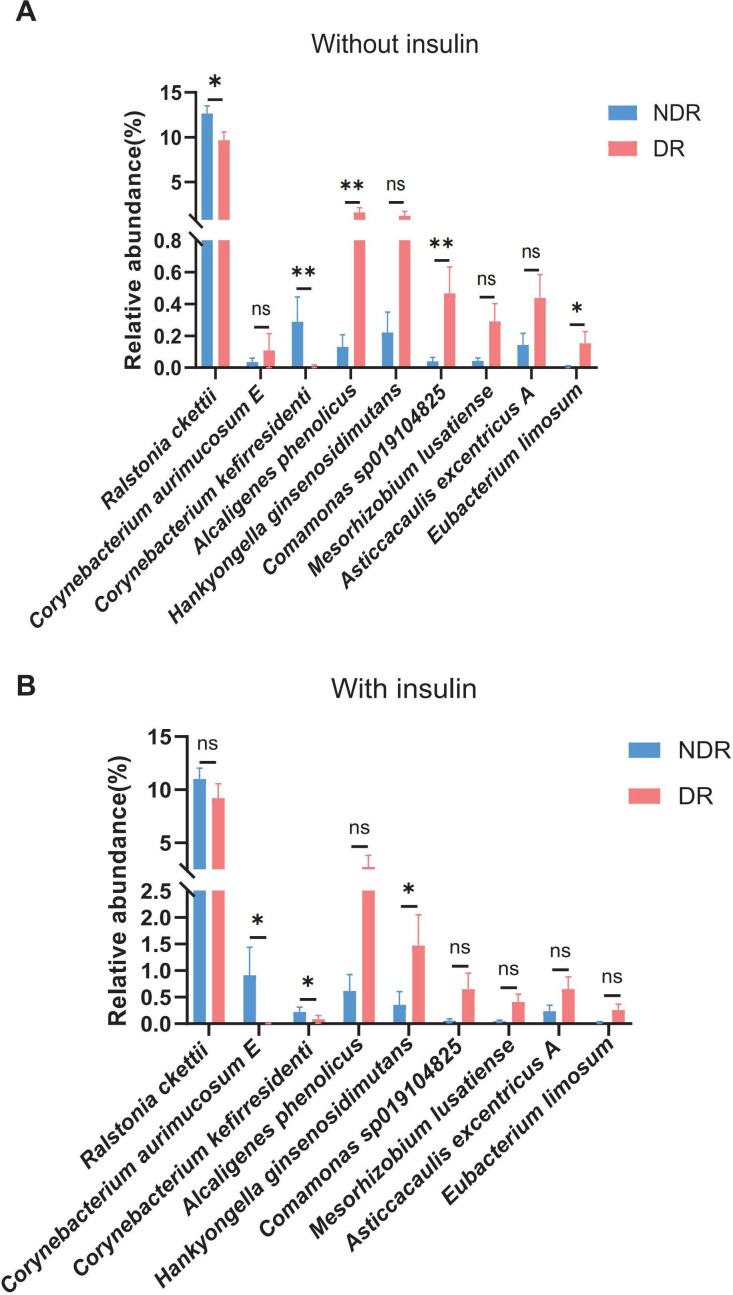
Evaluation of insulin effects on differential OSM species between DR and NDR. The differential species between DR and NDR identified by LEfSe were compared between the two groups using the KW test in patients with (**A**) and without (**B**) insulin treatment. *P＜0.05; **P＜0.01. ns, not significant.

The association between differential OSM taxa and clinical parameters was then analyzed using the Spearman rank-sum test (Tables S3 and S4) for continuous variables and the Wilcoxon rank-sum test for sex. The results of the Spearman analysis are shown for differential OSM genera and species with a correlation coefficient *r* > 0.3 in Fig. 6. For blood glucose-related parameters, HbA1c positively correlated with two genera, *Acinetobacter* and *Achromobacter*, and two species, *Acinetobacter johnsonii* and *Achromobacter xylosoxidans*, with the most significant positive correlation found in the genus *Acinetobacter* and its species *Acinetobacter johnsonii* (*r* = 0.35 and 0.41, respectively; Fig. 7; Tables S3 and S4). ROC curve analysis showed that *Acinetobacter johnsonii* gave an AUC value of 0.702 in predicting an HbA1c level ≥6.5% (Fig. S4). The genera *Brevundimonas* and *Achromobacter* and species *Achromobacter xylosoxidans* showed significant positive correlations with FBG. In contrast, HbA1c negatively correlated with one fungal genus, *Malassezia*; five bacterial genera, *Bifidobacterium, Lawsonella, Veillonella, Gordonia, and Clostridium*; one fungal species, *Malassezia restricta,* and nine bacterial species, such as *Cutibacterium granulosum, Clostridium neonatale,* and *Bifidobacterium bifidum*. These taxa (except the genus *Malassezia* and its species *Malassezia restricta*, and two bacterial species, *Paracoccus marinus* and *Cutibacterium granulosum*) also showed significant negative associations with FBG.

For kidney function-related parameters shown in Fig. 6 with *r* > 0.3, Cys-C negatively correlated with two genera *Rhizorhabdus* and *Achromobacter* and four species *Corynebacterium kefirresidentii*, *Aquabacterium sp913773725*, *Achromobacter xylosoxidans*, and *Achromobacter xylosoxidans*. UA negatively correlated with *Rhizorhabdus* and one of its species *Rhizorhabdus sp018006725*. Urea negatively correlated with *Rhizorhabdus sp018006725.* CREA negatively correlated with the genus *Brevundimonas*.

As for blood cell and immune function-related parameters shown in Fig. 6 with *r* > 0.3, RBP negatively correlated with the genus *Rhizorhabdus* and three species *Corynebacterium kefirresidentii, Corynebacterium simulans,* and *Rhizorhabdus sp018006725.* DBIL positively correlated with the genus *Deinococcus* and three *species Corynebacterium kefirresidentii*, *Corynebacterium simulans,* and *Rhizorhabdus sp018006725.* Three *g*enera *Bifidobacterium*, *Lawsonella,* and *Gordonia,* as well as five species *Lawsonella clevelandensis A*, *Gordonia bronchialis*, *Janibacter sp024362365, Bifidobacterium infantis, and Corynebacterium simulans,* positively correlated with both IBIL and TBIL*.* In addition*, Corynebacterium kefirresidentii* also negatively correlated with TBIL; in contrast, *Achromobacter* and two of its species, *Achromobacter xylosoxidans and Aquabacterium sp913773725,* negatively correlated with both IBIL and TBIL. NLR negatively correlated with two genera, *Afipia* and *Veillonella* and two *Corynebacterium* species *Corynebacterium kefirresidentii* and *Corynebacterium simulans*. β2-MG negatively correlated with the genus *Afipia* and two species *Cutibacterium granulosum* and *Corynebacterium kefirresidentii*. HBG and HCT positively correlated with the genus *Afipia* and three species *Corynebacterium kefirresidentii*, *Corynebacterium simulans*, and *Clostridium neonatale.* RBC positively correlated with *Afipia* and *Cutibacterium granulosum*. ALB positively correlated with the genera *Afipia* and *Clostridium* and negatively correlated with the genus *Acinetobacter* and its species *Acinetobacter johnsonii*.

In addition, the genus *Deinococcus* correlated positively with age, and species *Listeria monocytogenes* increased significantly in females (FDR = 0.021) but remained undetected in males.

### Potential effects of insulin treatment on OSM

Of the DM patients in our current cohort, 32.81% received insulin injection therapy. We thus explored its potential effects on the OSM. The abundances of differential species between DR and NDR identified by LEfSe analysis were further examined in patients according to their insulin injection status (Fig. 7). It was noted that the *Methylobacterium jeotgali* showed a significantly higher abundance in NDR than DR only in DM patients receiving insulin therapy, but not in those without treatment**.** Such a finding was further supported by logistic regression analysis, which suggested a remarkable interaction between insulin therapy and retinopathy status in DM patients (*P* < 0.05).

## DISCUSSION

DR is a chronic multifactorial complication of DM (26, 27). Recent studies based on 16S rRNA gene amplicon sequencing have shown that OSM dysbiosis may contribute to the occurrence and development of DR 12, 18. Our current study provides the first species-resolved OSM profiles in NC and DM with or without retinopathy using 2bRAD-M sequencing. The species-level changes in OSM associated with DM and DR are essential to better understand its role in these diseases.

The improved resolution at the species level in our current study might allow a more accurate evaluation of the OSM diversity. Previous studies based on 16S rRNA gene amplicon sequencing have reported inconsistent findings on the alpha diversity in DM. The species-resolved analysis in our 2bRAD-M sequencing study showed no significant changes in the OSM alpha diversity in DM (NDR or DR), consistent with Suwajanakorn’s 16S rRNA study (18). Significant differences were observed in the beta diversity of OSM in DR compared to NC or NDR. Notably, DR OSM showed high interindividual heterogeneity with more differentially enriched taxa, which is consistent with previous studies (14, 18). These findings could underscore a higher extent of OSM dysbiosis in DR than in NDR.

Our findings showed substantial OSM changes at the species level in DM, especially in DR patients, which were highly correlated with disease-related blood test parameters. Notably, Pseudomonadota and multiple taxa, including genera and species belonging to Pseudomonadota, showed the most substantial enrichment in DM, which were primarily attributable to DR. *Pseudomonas aeruginosa* and its genus *Pseudomonas* were the most significantly increased in DM, especially in DR. *Pseudomonas aeruginosa* is a widespread gram-negative, ubiquitous environmental and opportunistic pathogen capable of causing keratitis, blepharitis, conjunctivitis, and blepharoconjunctivitis (28–30). *Pseudomonas aeruginosa* plays a crucial role in diabetic foot infections, and its presence can be detected in infected incisions in patients (31). Our findings thus supported a strong link between the opportunistic pathogen and DR. In contrast, multiple taxa belonging to the Actinomycetota, such as commensals *Bifidobacterium bifidum* and *Bifidobacterium infantis*, substantially decreased in DM, especially in DR patients, and negatively correlated with FBG and HbA1c. Such findings suggested that Actinomycetota could be part of the healthy OSM, and its reduction could be involved in the development of the diseases. A pilot study by Chisari et al. reported that supplementation with *Bifidobacterium lactis* and *Bifidobacterium bifidum* improved the effect of artificial tear in reducing damage to the ocular surface, particularly in cases of dry eye disease (32). Notably, fungi were also found in the OSM. Importantly, *Malassezia restricta*, known to be part of the normal healthy skin microbiota (33), was predominant in NC and significantly decreased in DM/DR, suggesting that it could also be part of the normal OSM and warranting further study of its role in the diseases.

In our current study, *Sphingomonas paucimobilis* was identified as a prominent component of the OSM. While its high abundance is relatively uncommon in some previous reports, we implemented rigorous quality control measures to ensure these findings were not artifacts of contamination. The presence of *Sphingomonas paucimobilis* in the OSM is supported by its taxonomic and clinical context. Formerly classified as *Pseudomonas paucimobilis*, it belongs to a genus frequently documented as a core member of the normal OSM. Recent studies have identified *Sphingomonas paucimobilis* on the healthy ocular surface and have noted its potential role in bacterial contamination of medical instruments, such as intravitreal needles (34). Given our stringent physical and computational decontamination measures, the observed abundance of *Sphingomonas paucimobilis* probably represents a genuine biological signature within our study cohort. Further studies thus warrant analyzing the abundance of *Sphingomonas paucimobilis* in independent cohorts.

Moreover, our results suggested the diagnostic potential of the OSM species in predicting DM and DR, especially *Gordonia bronchialis* for DM diagnosis. *Gordonia bronchialis*, which was decreased in DM and DR, is a slow-growing, gram-positive aerobic bacillus belonging to Actinomycetota, one of the major phyla decreased in DR in our current study. The exact role of *Gordonia bronchialis* in DM and DR remains to be elucidated in further study.

Our current study showed significant yet divergent associations between the OSM and disease-related clinical parameters. Recent studies have shown that HbA1c and other biochemical parameters are associated with specific gut bacteria (35). Our current study found that a set of differential OSM species correlated with FBG and HbA1c mostly showed inverted correlations with IBIL and TBIL. Bilirubin is a byproduct of the typical breakdown of red blood cells, and studies have found that elevated TBIL and IBIL levels are associated with a lower risk of developing DR and diabetic kidney disease (DKD) (36). Notably, *Acinetobacter johnsonii* was the most positively correlated with elevated HbA1c levels. *Acinetobacter johnsonii* has been reported to be substantially altered in the OSM of dry eye patients and to be associated with human tear proteins (37). Moreover, studies have shown the presence of Acinetobacter in the serum of diabetic patients, along with elevated levels of inflammatory cytokines, suggesting a link between *Acinetobacter* and the chronic inflammation associated with DM (38). In addition, another set of differential OSM species was negatively correlated with Cys-C, UA, CREA, β2-MG, RBP, and NLR, all of which were elevated in DR in our cohort. These clinical parameters have been associated with an increased risk of DKD (39–44). In particular, Cys-C increased significantly in DR and decreased in NDR compared to NC in our cohort, in line with a previous study reporting it as a marker of DR (45). *Corynebacterium kefirresidentii,* a common bacterium found on human skin and belonging to Actinomycetota (46), decreased in DR of our cohort and was the species most negatively correlated with Cys-C. Although the underlying mechanisms remain largely unclear, such findings underline the role of the OSM in the relationship between DR and DKD (47). Specifically, elevated Cys-C reflects not only renal impairment but also the systemic inflammatory state and microvascular damage that characterize advanced stages of DR, such as PDR. Similarly, β2-MG has been identified as a reliable biomarker for diabetic microvascular complications. Such findings suggested that the OSM changes could be primarily due to uncontrolled hyperglycemia, which alters tear film chemistry. This metabolic shift could be coupled with secondary ocular surface impairment, creating a unique niche that favors the overgrowth of specific taxa in response to the escalating systemic microvascular burden associated with DR progression.

Our current study also had limitations. First, it did not include treatment-naïve DM patients or those who had undergone vitreoretinal surgery. Nevertheless, our analysis suggested that insulin treatment might not strongly affect most of the differential OSM species between DR and NDR and could influence the abundance of a specific species, *Methylobacterium jeotgali*. Second, larger sample sizes in future studies should enable investigation of the OSM of DR across different disease stages. Furthermore, our study did not explicitly analyze the effects of contact lenses, glasses, or eye drops on the OSM. Previous studies have suggested that contact lens wear can alter the microbial composition of the OSM (48). Although our rigorous exclusion of symptomatic ocular diseases may have filtered out frequent users of medicated eye drops, the potential influence of these factors on the OSM cannot be ruled out entirely. Future studies with more granular inclusion criteria are warranted.

In conclusion, our 2bRAD-M sequencing study provided the first species-resolved OSM profiles and revealed dramatic changes in DM, especially in DR patients, compared to NC, and their association with disease-related clinical parameters. Nevertheless, further studies are warranted to thoroughly explore the role of OSM in DM and DR.

## Data Availability

The 2bRAD-M sequencing raw data in the current study have been deposited in the Genome Sequence Archive at the National Genomics Data Center, Beijing Institute of Genomics, Chinese Academy of Sciences/China National Center for Bioinformation (GSA: PRJCA037496) and are publicly accessible at https://ngdc.cncb.ac.cn/gsa.

## SUPPLEMENTAL MATERIAL

Supplemental Material msphere.00003-26-s0001.pdf Fig. S1 to S4; Tables S1 to S4.

ASM does not own the copyrights to Supplemental Material that may be linked to, or accessed through, an article. The authors have granted ASM a non-exclusive, world-wide license to publish the Supplemental Material files. Please contact the corresponding author directly for reuse.
