# Supplementary material for: Association between ocular surface microbiota species and type 2 diabetes mellitus with or without retinopathy and related clinical parameters
Source: mSphere. 2026 May 12;11(6):e00003-26. doi: 10.1128/msphere.00003-26 (PMC13317193; doi:10.1128/msphere.00003-26)
Supplement: Supplemental Material — Fig. S1 to S4; Tables S1 to S4. [file msphere.00003-26-s0001.pdf]

Supplementary Material

Species-solved profiling and clinical correlations of ocular surface microbiota in diabetic retinopathy

Supplementary Figures

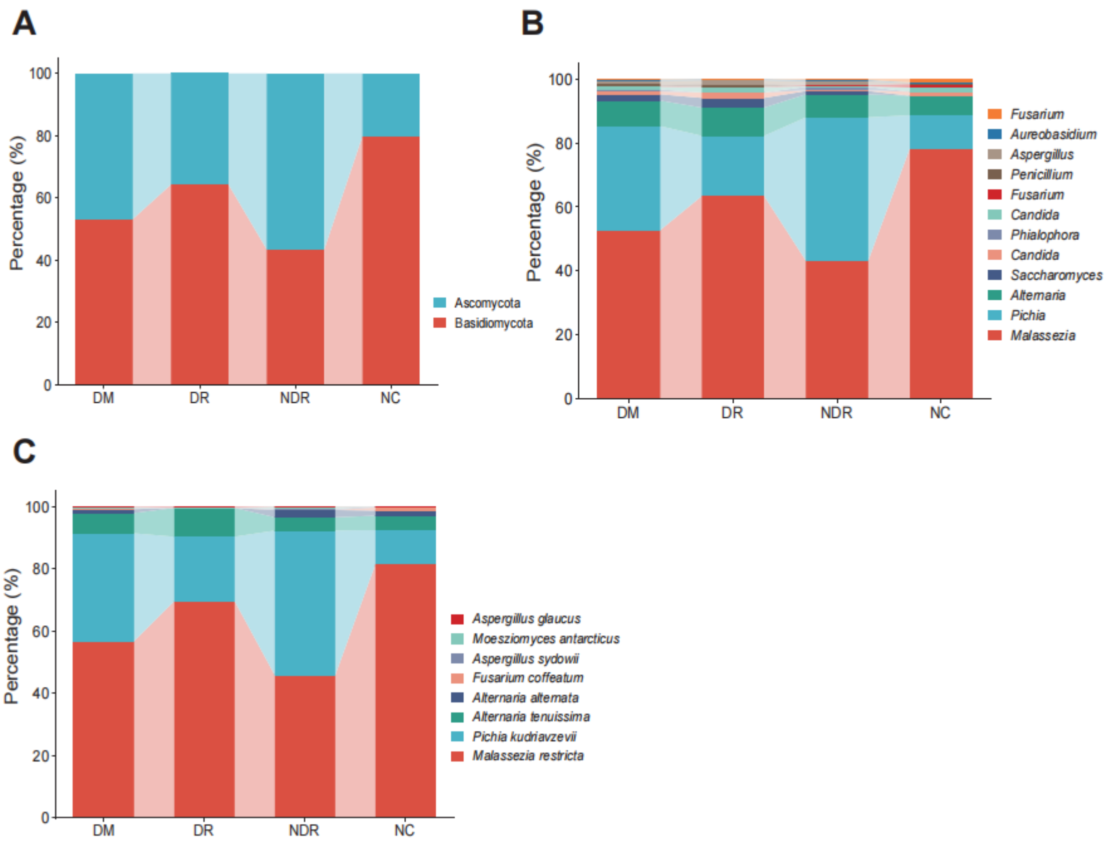

Supplementary Figure 1. Fungal abundance and composition among all groups at the phylum(**A**), genus(**B**), and species levels(**C**).

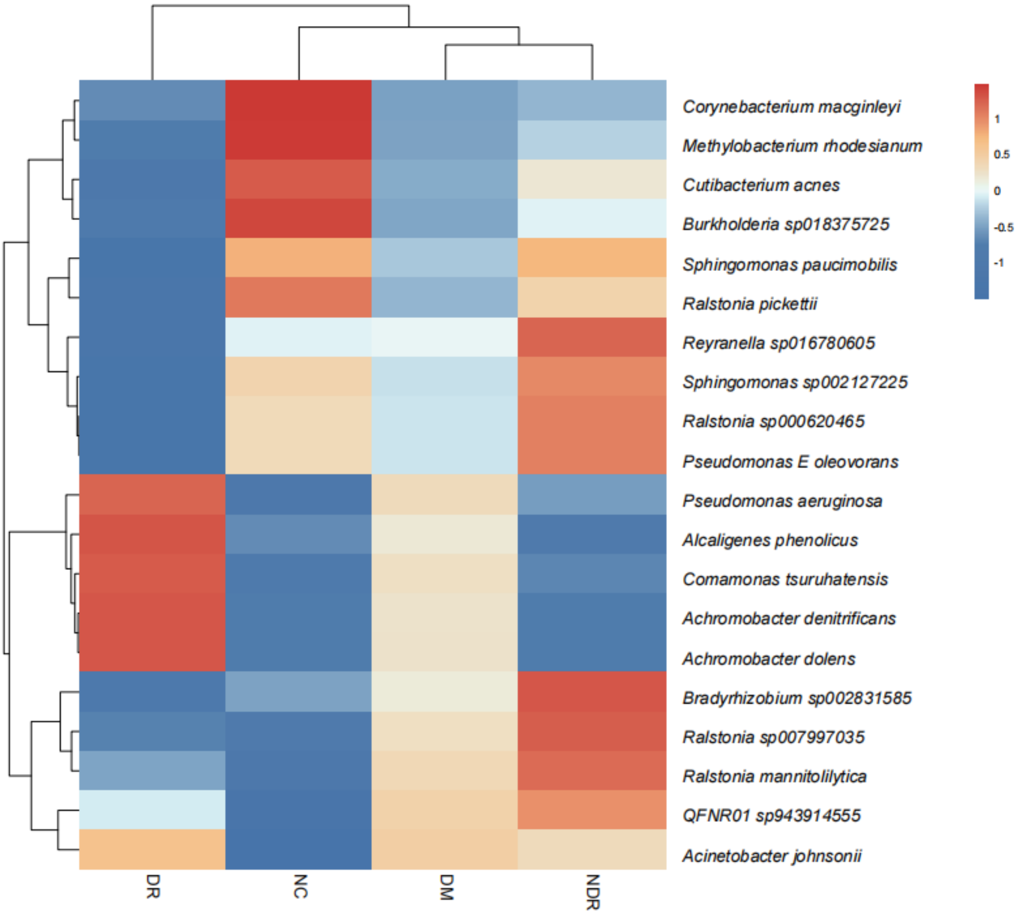

Supplementary Figure 2. Cluster heatmap showing the z-score transformed relative abundance of the top 20 species across all groups.

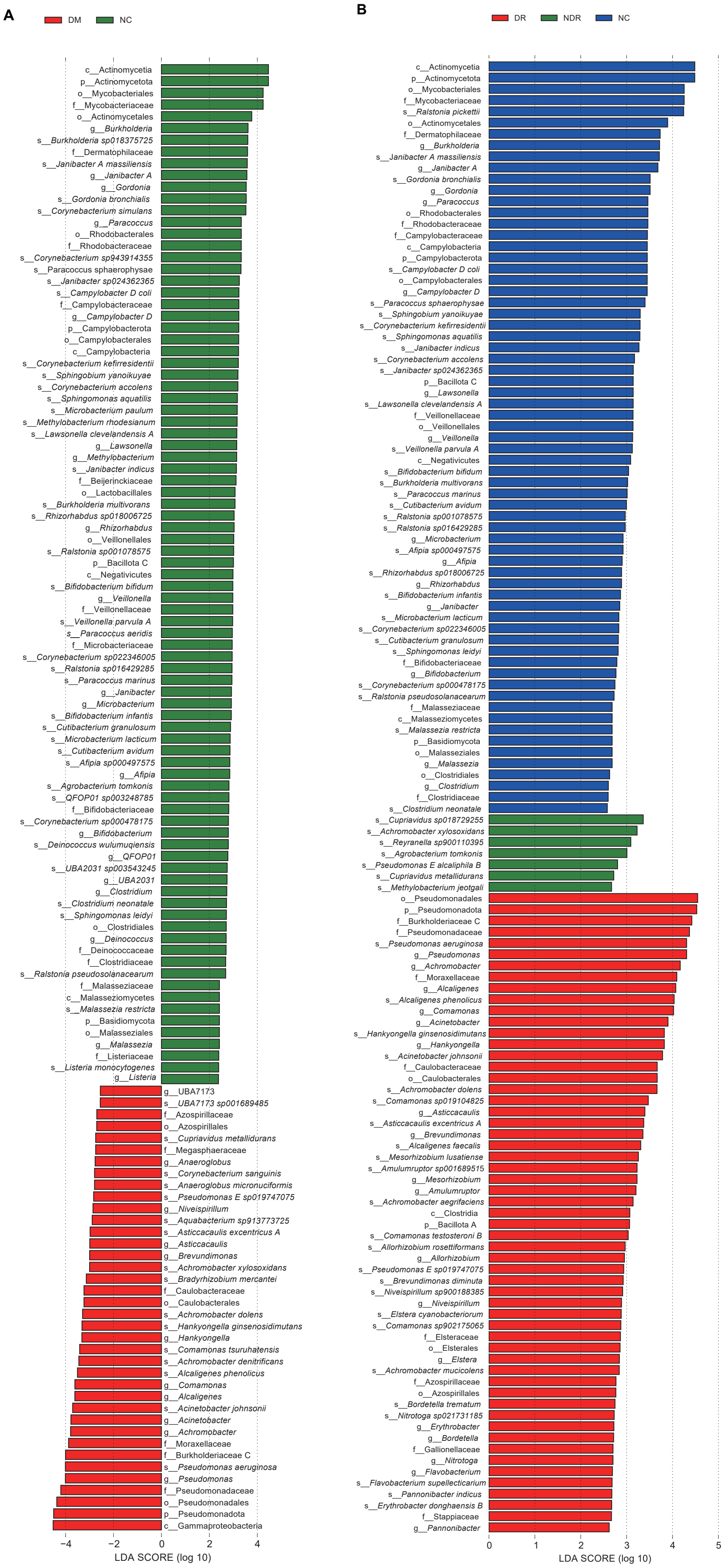
A
DM
NC
c__Actinomycetia
p__Actinomycetota
o__Mycobacteriales
f__Mycobacteriaceae
o__Actinomycetales
g__Burkholderia
s__Burkholderia sp018375725
f__Dermatophilaceae
s__Janibacter A massiliensis
g__Janibacter A
g__Gordonia
s__Gordonia bronchialis
s__Corynebacterium simulans
g__Paracoccus
o__Rhodobacterales
f__Rhodobacteraceae
s__Corynebacterium sp943914355
s__Paracoccus sphaerophysae
s__Janibacter sp024362365
s__Campylobacter D coli
f__Campylobacteraceae
g__Campylobacter D
p__Campylobacterota
o__Campylobacterales
c__Campylobacteria
s__Corynebacterium kefirresidentii
s__Sphingobium yanoikuyae
s__Corynebacterium accolens
s__Sphingomonas aquatilis
s__Microbacterium paulum
s__Methylobacterium rhodesianum
s__Lawsonella clevelandensis A
g__Lawsonella
g__Methylobacterium
s__Janibacter indicus
f__Beijerinckiaceae
o__Lactobacillales
s__Burkholderia multivorans
s__Rhizorhabdus sp018006725
g__Rhizorhabdus
o__Veillonellales
s__Ralstonia sp001078575
p__Bacillota C
c__Negativicutes
s__Bifidobacterium bifidum
g__Veillonella
f__Veillonellaceae
s__Veillonella parvula A
s__Paracoccus aeridis
f__Microbacteriaceae
s__Corynebacterium sp022346005
s__Ralstonia sp016429285
s__Paracoccus marinus
g__Janibacter
g__Microbacterium
s__Bifidobacterium infantis
s__Cutibacterium granulosum
s__Microbacterium lacticum
s__Cutibacterium avidum
s__Afipia sp000497575
g__Afipia
s__Agrobacterium tomkonis
s__QFOP01 sp003248785
f__Bifidobacteriaceae
s__Corynebacterium sp000478175
g__Bifidobacterium
s__Deinococcus wulumuqiensis
g__QFOP01
s__UBA2031 sp003543245
g__UBA2031
g__Clostridium
s__Clostridium neonatale
s__Sphingomonas leidyi
o__Clostridiales
g__Deinococcus
f__Deinococcaceae
f__Clostridiaceae
s__Ralstonia pseudosolanacearum
f__Malasseziaceae
c__Malasseziomycetes
s__Malassezia restricta
p__Basidiomycota
o__Malasseziales
g__Malassezia
f__Listeriaceae
s__Listeria monocytogenes
g__Listeria
g__UBA7173
s__UBA7173 sp001689485
f__Azospirillaceae
o__Azospirillales
s__Cupriavidus metallidurans
f__Megasphaeraceae
g__Anaeroglobus
s__Corynebacterium sanguinis
s__Anaeroglobus micronuciformis
s__Pseudomonas E sp019747075
g__Niveispirillum
s__Aquabacterium sp913773725
s__Asticcacaulis excentricus A
g__Asticcacaulis
g__Brevundimonas
s__Achromobacter xylosoxidans
s__Bradyrhizobium mercantei
f__Caulobacteraceae
o__Caulobacterales
s__Achromobacter dolens
s__Hankyongella ginsenosidimutans
g__Hankyongella
s__Comamonas tsuruhatensis
s__Achromobacter denitrificans
s__Alcaligenes phenolicus
g__Comamonas
g__Alcaligenes
s__Acinetobacter johnsonii
g__Acinetobacter
g__Achromobacter
f__Moraxellaceae
f__Burkholderiaceae C
s__Pseudomonas aeruginosa
g__Pseudomonas
f__Pseudomonadaceae
o__Pseudomonadales
p__Pseudomonadota
c__Gammaproteobacteria
−4
−2
0
2
4
LDA SCORE (log 10)
B
DR
NDR
NC
c__Actinomycetia
p__Actinomycetota
o__Mycobacteriales
f__Mycobacteriaceae
s__Ralstonia pickettii
o__Actinomycetales
f__Dermatophilaceae
g__Burkholderia
s__Janibacter A massiliensis
g__Janibacter A
s__Gordonia bronchialis
g__Gordonia
g__Paracoccus
o__Rhodobacterales
f__Rhodobacteraceae
f__Campylobacteraceae
c__Campylobacteria
p__Campylobacterota
s__Campylobacter D coli
o__Campylobacterales
g__Campylobacter D
s__Paracoccus sphaerophysae
s__Sphingobium yanoikuyae
s__Corynebacterium kefirresidentii
s__Sphingomonas aquatilis
s__Janibacter indicus
s__Corynebacterium accolens
s__Janibacter sp024362365
p__Bacillota C
g__Lawsonella
s__Lawsonella clevelandensis A
f__Veillonellaceae
o__Veillonellales
g__Veillonella
s__Veillonella parvula A
c__Negativicutes
s__Bifidobacterium bifidum
s__Burkholderia multivorans
s__Paracoccus marinus
s__Cutibacterium avidum
s__Ralstonia sp001078575
s__Ralstonia sp016429285
g__Microbacterium
s__Afipia sp000497575
g__Afipia
s__Rhizorhabdus sp018006725
g__Rhizorhabdus
s__Bifidobacterium infantis
g__Janibacter
s__Microbacterium lacticum
s__Corynebacterium sp022346005
s__Cutibacterium granulosum
s__Sphingomonas leidyi
f__Bifidobacteriaceae
g__Bifidobacterium
s__Corynebacterium sp000478175
s__Ralstonia pseudosolanacearum
f__Malasseziaceae
c__Malasseziomycetes
s__Malassezia restricta
p__Basidiomycota
o__Malasseziales
g__Malassezia
o__Clostridiales
g__Clostridium
f__Clostridiaceae
s__Clostridium neonatale
s__Cupriavidus sp018729255
s__Achromobacter xylosoxidans
s__Reyranella sp900110395
s__Agrobacterium tomkonis
s__Pseudomonas E alcaliphila B
s__Cupriavidus metallidurans
s__Methylobacterium jeotgali
o__Pseudomonadales
p__Pseudomonadota
f__Burkholderiaceae C
f__Pseudomonadaceae
s__Pseudomonas aeruginosa
g__Pseudomonas
g__Achromobacter
f__Moraxellaceae
g__Alcaligenes
s__Alcaligenes phenolicus
g__Comamonas
g__Acinetobacter
s__Hankyongella ginsenosidimutans
g__Hankyongella
s__Acinetobacter johnsonii
f__Caulobacteraceae
o__Caulobacterales
s__Achromobacter dolens
s__Comamonas sp019104825
g__Asticcacaulis
s__Asticcacaulis excentricus A
g__Brevundimonas
s__Alcaligenes faecalis
s__Mesorhizobium lusatiense
s__Amulumruptor sp001689515
g__Mesorhizobium
g__Amulumruptor
s__Achromobacter aegrifaciens
c__Clostridia
p__Bacillota A
s__Comamonas testosteroni B
s__Allorhizobium rosettiformans
g__Allorhizobium
s__Pseudomonas E sp019747075
s__Brevundimonas diminuta
s__Niveispirillum sp900188385
g__Niveispirillum
s__Elstera cyanobacteriorum
s__Comamonas sp902175065
f__Elsteraceae
o__Elsterales
g__Elstera
s__Achromobacter mucicolens
f__Azospirillaceae
o__Azospirillales
s__Bordetella trematum
s__Nitrotoga sp021731185
g__Erythrobacter
g__Bordetella
f__Gallionellaceae
g__Nitrotoga
g__Flavobacterium
s__Flavobacterium supellecticarium
s__Pannonibacter indicus
s__Erythrobacter donghaensis B
f__Stappiaceae
g__Pannonibacter
0
1
2
3
4
5
LDA SCORE (log 10)

C

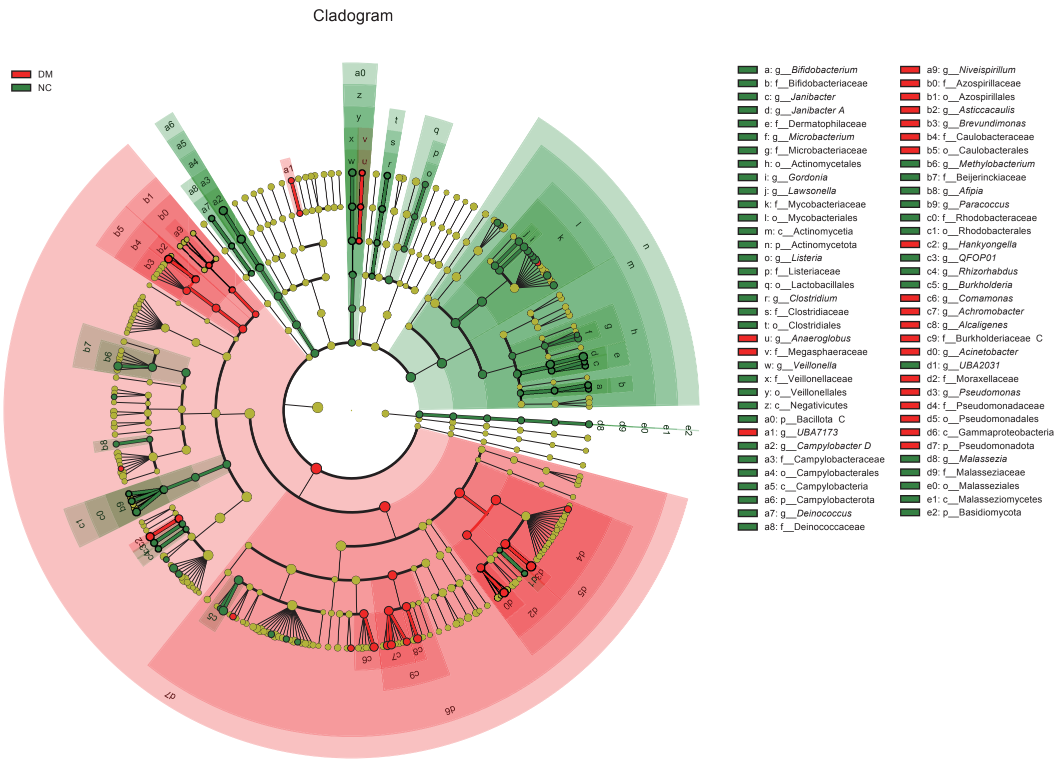

D

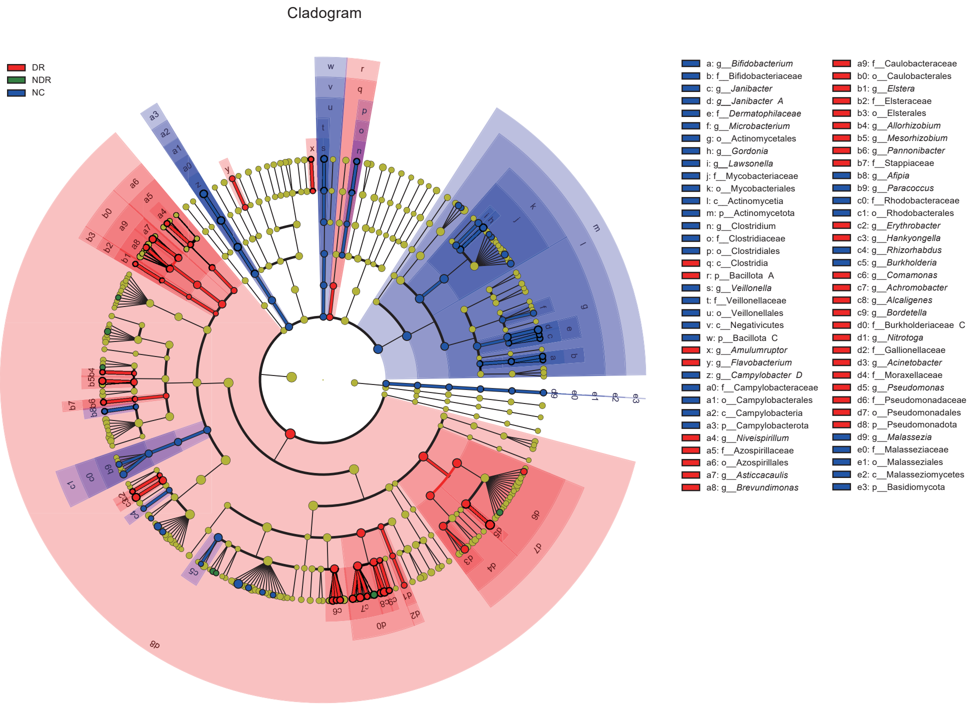

Supplementary Figure 3. Differentially abundant taxa among groups identified by LEfSe. LDA scores (**A-B**) and cladograms (**C-D**) are shown for comparisons between DM and NC and between DR, NDR, and NC.

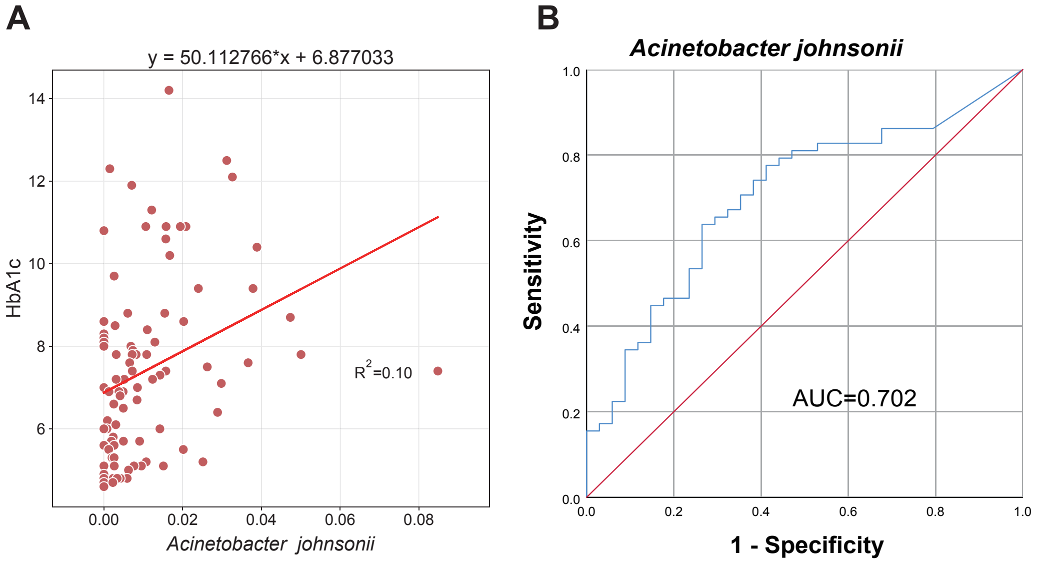

Supplementary Figure 4. The performance of *Acinetobacter johnsonii* in predicting the HbA1c level. **(A)** Linear regression analysis of the HbA1c level and relative abundance of *Acinetobacter johnsonii*. **(B)** ROC analysis of *Acinetobacter johnsonii* in predicting an HbA1c level ⩾6.5%.

Supplementary Table 1. Adaptors and primers used for 2bRAD-M library preparation.

Adaptors	Sequence (5′ to 3′)
Adap-1 sense	ACACTCTTTCCCTACACGACGCTCTTCCGATCTNN
Adap-1 antisense	AGATCGGAAGAGC
Adap-2 sense	GTGACTGGAGTTCAGACGTGTGCTCTTCCGATCTNN
Adap-2 antisense	AGATCGGAAGAGC
Primer1	ACACTCTTTCCCTACACGACGCT
Primer2	GTGACTGGAGTTCAGACGTGTGCT
5UDI Primer	AATGATACGGCGACCACCGAGATCTACACXXXXXXXXAC ACTCTTTCCCTACACGACGCTCTTCCGATCT
7UDI Primer	CAAGCAGAAGACGGCATACGAGATXXXXXXXXGTGACTG GAGTTCAGACGTGTGCTCTTCCGATCT

Supplementary Table 2. Quality control summary of sequencing data: number of raw reads, enzyme reads, clean reads and percent.

Sample	Raw_Reads	Enzymatic_Reads	Clean_Reads	Percentage of Clean reads
DR_1	9626309	5685715	5415714	56.26%

DR_2	7749414	5449798	5206124	67.18%
DR_3	6227582	3279516	3125789	50.19%
DR_4	6384583	5068131	4837309	75.77%
DR_5	7195412	4701478	4474109	62.18%
DR_6	4608087	4608087	4387880	62.19%
DR_7	5278815	5278815	5028731	71.27%
DR_8	5278815	4800459	4574531	69.49%
DR_9	3856031	3856031	3675283	59.37%
DR_10	4451040	4451040	4251944	66.20%
DR_11	6802178	5206170	5206170	72.96%
DR_12	7194976	7194976	4624828	64.28%
DR_13	6091478	3702294	3530719	57.96%
DR_14	7537637	4777588	4556190	60.45%
DR_15	6328878	6328878	6328878	64.42%
DR_16	7536193	7536193	7536193	65.95%
DR_17	6352268	4161816	3966000	62.43%
DR_18	6800495	4257968	4068290	59.82%
DR_19	6157779	3982905	3788217	61.52%
DR_20	7749414	5017173	4801715	70.91%
DR_21	6291304	3895741	3895741	58.99%
DR_22	6912277	4966654	4733847	68.48%
DR_23	6235120	3593842	3421162	54.87%
DR_24	7007761	5088433	4849514	69.20%
DR_25	6247369	3757870	3562932	66.85%
DR_26	7462386	5239460	4988440	66.85%
DR_27	6428531	4338609	4112670	63.98%
DR_28	6182285	4668267	4443771	71.88%
DR_29	6303763	4492789	4290591	68.06%
DR_30	8064853	5285210	5025583	50.19%
DR_31	8064853	4892038	4641365	60.58%
NDR_1	7422398	4842094	4599203	61.96%
NDR_2	7693884	6134559	5840955	75.92%
NDR_3	6958359	4218075	4011749	57.65%
NDR_4	7282504	4824756	4590024	63.03%
NDR_5	6704287	4414694	4203886	62.70%
NDR_6	7041516	4830291	4610029	65.47%
NDR_7	8834852	6057019	5781553	65.44%
NDR_8	750170	4736624	4511539	60.14%
NDR_9	7985175	4663652	4440910	55.61%
NDR_10	6836847	5146156	4895349	71.60%
NDR_11	6464959	575214	4355175	67.37%
NDR_12	8594286	6001252	5716009	66.51%
NDR_13	8342138	5984056	5696734	68.29%
NDR_14	6263782	4609717	4398736	70.22%

NDR_15	8845708	5743756	5485488	62.01%
NDR_16	7705441	5159084	4865780	66.37%
NDR_17	7419034	5384788	5129725	69.14%
NDR_18	7419034	5159084	4865780	66.37%
NDR_19	6903016	5422113	5159750	74.75%
NDR_20	8073899	5423359	5189000	64.27%
NDR_21	9047616	6305812	6007652	66.40%
NDR_22	7356653	5219630	4996995	67.92%
NDR_23	8940883	6656113	6337494	70.88%
NDR_24	9262133	6414887	6165364	66.57%
NDR_25	7219842	6414887	6165364	66.57%
NDR_26	7469223	5465735	5211789	69.78%
NDR_27	7806704	5602581	5341909	68.43%
NDR_28	7940487	5025226	4804186	60.50%
NDR_29	8800627	5933480	5653187	64.24%
NDR_30	8197449	4218075	4011749	57.65%
NDR_31	7738491	5416842	5183703	63.24%
NDR_32	9367586	5139546	4897125	63.28%
NDR_33	7422940	5139546	4897125	63.28%
NC_1	7923194	5778089	5507061	69.51%
NC_2	7042121	3594798	3424114	48.62%
NC_3	6862078	3710361	3546494	51.68%
NC_4	7512838	4550297	4323625	57.55%
NC_5	7416896	3921424	3751689	50.58%
NC_6	6888314	3973724	3780542	54.88%
NC_7	8186212	5131278	4913961	60.03%
NC_8	6732815	4170534	3980291	59.12%
NC_9	7258102	4399234	4194569	57.79%
NC_10	6703597	4172586	3958593	59.05%
NC_11	6169953	4156217	3922381	63.57%
NC_12	6650795	4262627	3991874	60.02%
NC_13	6637891	4633544	4378606	65.96%
NC_14	6490768	4116130	3915641	60.33%
NC_15	6542092	4443272	4182019	63.92%
NC_16	6314092	4286654	4043073	64.03%
NC_17	6334146	3891512	3657805	57.75%
NC_18	6415664	4567165	4316507	67.28%
NC_19	6149135	4157822	3918231	63.72%
NC_20	6157237	4274406	3996706	64.91%
NC_21	6432891	4751341	4488588	69.78%
NC_22	6361885	4472866	4243440	66.70%
NC_23	7585153	5191777	4874805	64.27%
NC_24	6082680	4341900	4094693	67.32%
NC_25	6870259	4899195	4597950	66.93%

NC_26	6447842	4663548	4393029	68.13%
NC_27	6834310	4621111	4354210	63.71%
NC_28	7402603	5280881	4952993	66.91%

Supplementary Table 3. Correlation between differential genera and clinical parameters.

No.	Genus	Variable	*r*	*P*
1	*Deinococcus*	age	0.318	0.002
2	*Malassezia*	HbA1c	-0.401	<0.001
3	*Clostridium*	HbA1c	-0.402	<0.001
4	*Bifidobacterium*	HbA1c	-0.366	<0.001
5	*Janibacter*	HbA1c	-0.274	0.008
6	*Veillonella*	HbA1c	-0.391	<0.001
7	*Lawsonella*	HbA1c	-0.342	0.001
8	*Gordonia*	HbA1c	-0.455	<0.001
9	*Achromobacter*	HbA1c	0.253	0.015
10	*Acinetobacter*	HbA1c	0.347	0.001
11	*Alcaligenes*	HbA1c	0.223	0.033
12	*Hankyongella*	HbA1c	0.287	0.005
13	*Clostridium*	FBG	-0.313	0.002
14	*Bifidobacterium*	FBG	-0.259	0.013
15	*Janibacter*	FBG	-0.261	0.012
16	*Veillonella*	FBG	-0.323	0.002
17	*Methylobacterium*	FBG	-0.276	0.008
18	*Lawsonella*	FBG	-0.269	0.009
19	*Gordonia*	FBG	-0.359	<0.001
20	*Janibacter A*	FBG	-0.298	0.004
21	*Achromobacter*	FBG	0.330	0.001
22	*Alcaligenes*	FBG	0.226	0.030

23	*Comamonas*	FBG	0.259	0.013
24	*Hankyongella*	FBG	0.297	0.004
25	*Brevundimonas*	FBG	0.303	0.003
26	*Asticcacaulis*	FBG	0.249	0.017
27	*Bordetella*	FBG	0.248	0.017
28	*Flavobacterium*	FBG	0.227	0.030
29	*Rhizorhabdus*	Cys-C	-0.227	0.030
30	*Achromobacter*	Cys-C	-0.228	0.029
31	*Elstera*	Cys-C	0.219	0.036
32	*Listeria*	UA	-0.212	0.042
33	*Microbacterium*	UA	-0.215	0.040
34	*Rhizorhabdus*	UA	-0.215	0.039
35	*Burkholderia*	UA	-0.240	0.021
36	*Amulumruptor*	UA	0.277	0.008
37	*Erythrobacter*	UA	0.255	0.014
38	*Nitrotoga*	UA	0.209	0.046
39	*Listeria*	urea	-0.273	0.008
40	*Elstera*	urea	0.210	0.044
41	*Listeria*	CREA	-0.223	0.033
42	*Brevundimonas*	CREA	-0.225	0.031
43	*Afipia*	β2-MG	-0.230	0.028
44	*Janibacter*	β2-MG	-0.265	0.011
45	*Campylobacter D*	β2-MG	-0.275	0.008
46	*Burkholderia*	β2-MG	-0.217	0.038
47	*Microbacterium*	RBP	-0.271	0.009
48	*Rhizorhabdus*	RBP	-0.320	0.002

49	*Janibacter A*	RBP	-0.225	0.031
50	*Burkholderia*	RBP	-0.207	0.047
51	*Alcaligenes*	RBP	0.211	0.044
52	*Deinococcus*	DBIL	0.262	0.012
53	*UBA7173*	DBIL	0.214	0.040
54	*Erythrobacter*	DBIL	-0.209	0.046
55	*Nitrotoga*	DBIL	-0.229	0.028
56	*Bifidobacterium*	IBIL	0.318	0.002
57	*Janibacter*	IBIL	0.280	0.007
58	*Lawsonella*	IBIL	0.300	0.004
59	*Campylobacter D*	IBIL	0.210	0.044
60	*Gordonia*	IBIL	0.254	0.015
61	*Burkholderia*	IBIL	0.237	0.023
62	*Bifidobacterium*	TBIL	0.336	0.001
63	*Janibacter*	TBIL	0.292	0.005
64	*Methylobacterium*	TBIL	0.246	0.018
65	*Lawsonella*	TBIL	0.324	0.002
66	*Campylobacter D*	TBIL	0.246	0.018
67	*Gordonia*	TBIL	0.245	0.019
68	*Burkholderia*	TBIL	0.274	0.008
69	*Achromobacter*	TBIL	-0.223	0.033
70	*Alcaligenes*	TBIL	-0.248	0.017
71	*Hankyongella*	TBIL	-0.224	0.032
72	*Asticcacaulis*	TBIL	-0.225	0.031
73	*Mesorhizobium*	TBIL	-0.272	0.009
74	*Erythrobacter*	TBIL	-0.237	0.023

75	*Afipia*	NLR	-0.263	0.011
76	*Microbacterium*	NLR	-0.217	0.038
77	*Veillonella*	NLR	-0.248	0.017
78	*Campylobacter D*	NLR	-0.249	0.017
79	*Niveispirillum*	NLR	0.220	0.035
80	*Elstera*	NLR	0.212	0.043
81	*Afipia*	HBG	0.311	0.003
82	*Paracoccus*	HBG	0.209	0.046
83	*Afipia*	RBC	0.315	0.002
84	*Janibacter A*	RBC	-0.209	0.045
85	*Afipia*	HCT	0.335	0.001
86	*Janibacter A*	HCT	-0.230	0.028
87	*Amulumruptor*	HCT	-0.212	0.043
88	*Clostridium*	ALB	0.246	0.018
89	*Afipia*	ALB	0.334	0.001
90	*Acinetobacter*	ALB	-0.217	0.038

Supplementary Table 4. Correlation between differential species and clinical parameters.

No.	Species	Variable	*r*	*P*
1	*Deinococcus wulumuqiensis*	age	0.225	0.031
2	*Bifidobacterium bifidum*	HbA1c	-0.409	<0.001
3	*Bifidobacterium infantis*	HbA1c	-0.341	0.001
4	*Janibacter indicus*	HbA1c	-0.259	0.013
5	*Janibacter sp024362365*	HbA1c	-0.374	<0.001
6	*Microbacterium lacticum*	HbA1c	-0.239	0.022

7	*Corynebacterium accolens*	HbA1c	-0.297	0.004
8	*Corynebacterium sanguinis*	HbA1c	0.246	0.018
9	*Corynebacterium sp943914355*	HbA1c	-0.225	0.031
10	*Gordonia bronchialis*	HbA1c	-0.467	<0.001
11	*Lawsonella clevelandensis A*	HbA1c	-0.343	0.001
12	*Cutibacterium avidum*	HbA1c	-0.237	0.023
13	*Cutibacterium granulosum*	HbA1c	-0.216	0.038
14	*Clostridium neonatale*	HbA1c	-0.316	0.002
15	*Veillonella parvula A*	HbA1c	-0.302	0.003
16	*Methylobacterium rhodesianum*	HbA1c	-0.221	0.034
17	*Agrobacterium tomkonis*	HbA1c	-0.218	0.037
18	*Afipia sp000497575*	HbA1c	-0.217	0.038
19	*Paracoccus marinus*	HbA1c	-0.376	<0.001
20	*Paracoccus sphaerophysae*	HbA1c	-0.292	0.005
21	*Hankyongella ginsenosidimutans*	HbA1c	0.287	0.005
22	*Sphingobium yanoikuyae*	HbA1c	-0.268	0.010
23	*Cupriavidus metallidurans*	HbA1c	0.261	0.012
24	*Ralstonia sp001078575*	HbA1c	-0.264	0.011
25	*Achromobacter aegrifaciens*	HbA1c	0.216	0.038
26	*Achromobacter xylosoxidans*	HbA1c	0.327	0.001
27	*Acinetobacter johnsonii*	HbA1c	0.407	<0.001
28	*Pseudomonas E sp019747075*	HbA1c	0.246	0.018
29	*Malassezia restricta*	HbA1c	-0.405	<0.001
30	*Bifidobacterium bifidum*	FBG	-0.375	<0.001
31	*Bifidobacterium infantis*	FBG	-0.207	0.048
32	*Janibacter indicus*	FBG	-0.227	0.030

33	*Janibacter sp024362365*	FBG	-0.297	0.004
34	*Janibacter A massiliensis*	FBG	-0.298	0.004
35	*Gordonia bronchialis*	FBG	-0.380	<0.001
36	*Lawsonella clevelandensis A*	FBG	-0.270	0.009
37	*Cutibacterium avidum*	FBG	-0.254	0.015
38	*Clostridium neonatale*	FBG	-0.224	0.032
39	*Anaeroglobus micronuciformis*	FBG	-0.246	0.018
40	*Veillonella parvula A*	FBG	-0.283	0.006
41	*Asticcacaulis excentricus A*	FBG	0.248	0.017
42	*Methylobacterium rhodesianum*	FBG	-0.282	0.006
43	*Paracoccus sphaerophysae*	FBG	-0.232	0.026
44	*Hankyongella ginsenosidimutans*	FBG	0.297	0.004
45	*Sphingomonas aquatilis*	FBG	-0.243	0.020
46	*Cupriavidus metallidurans*	FBG	0.280	0.007
47	*Ralstonia sp001078575*	FBG	-0.262	0.012
48	*Comamonas sp902175065*	FBG	0.265	0.011
49	*Comamonas tsuruhatensis*	FBG	0.224	0.032
50	*Achromobacter aegrifaciens*	FBG	0.224	0.032
51	*Achromobacter xylosoxidans*	FBG	0.415	<0.001
52	*Alcaligenes faecalis*	FBG	0.216	0.039
53	*Alcaligenes phenolicus*	FBG	0.208	0.046
54	*Bordetella trematum*	FBG	0.266	0.010
55	*Pseudomonas E sp019747075*	FBG	0.279	0.007
56	*Corynebacterium kefirresidentii*	Cys-C	-0.340	0.001
57	*Elstera cyanobacteriorum*	Cys-C	0.219	0.036
58	*Reyranella sp900110395*	Cys-C	-0.246	0.018

59	*Agrobacterium tomkonis*	Cys-C	-0.219	0.036
60	*Rhizorhabdus sp018006725*	Cys-C	-0.226	0.031
61	*Aquabacterium sp913773725*	Cys-C	-0.227	0.030
62	*Achromobacter xylosoxidans*	Cys-C	-0.207	0.048
63	*Amulumruptor sp001689515*	UA	0.237	0.023
64	*Erythrobacter donghaensis B*	UA	0.266	0.011
65	*Rhizorhabdus sp018006725*	UA	-0.214	0.041
66	*Sphingobium yanoikuyae*	UA	-0.211	0.043
67	*Sphingomonas aquatilis*	UA	-0.274	0.008
68	*Sphingomonas leidyi*	UA	-0.271	0.009
69	*Burkholderia sp018375725*	UA	-0.232	0.026
70	*Cupriavidus metallidurans*	UA	-0.250	0.016
71	*Ralstonia sp001078575*	UA	-0.225	0.031
72	*Achromobacter aegrifaciens*	UA	0.233	0.025
73	*Achromobacter dolens*	UA	0.216	0.039
74	*Nitrotoga sp021731185*	UA	0.209	0.046
75	*Corynebacterium kefirresidentii*	urea	-0.297	0.004
76	*Listeria monocytogenes*	urea	-0.277	0.007
77	*Elstera cyanobacteriorum*	urea	0.210	0.044
78	*Agrobacterium tomkonis*	urea	-0.247	0.018
79	*Sphingobium yanoikuyae*	urea	-0.264	0.011
80	*Sphingomonas aquatilis*	urea	-0.285	0.006
81	*Ralstonia pseudosolanacearum*	urea	-0.232	0.026
82	*Sphingomonas aquatilis*	CREA	-0.224	0.032
83	*Janibacter sp024362365*	β2-MG	-0.223	0.032
84	*Corynebacterium kefirresidentii*	β2-MG	-0.223	0.032

85	*Cutibacterium granulosum*	β2-MG	-0.236	0.024
86	*Campylobacter D coli*	β2-MG	-0.275	0.008
87	*Reyranella sp900110395*	β2-MG	-0.266	0.010
88	*Agrobacterium tomkonis*	β2-MG	-0.216	0.039
89	*Sphingobium yanoikuyae*	β2-MG	-0.239	0.022
90	*Sphingomonas aquatilis*	β2-MG	-0.272	0.009
91	*Sphingomonas leidyi*	β2-MG	-0.211	0.044
92	*Burkholderia sp018375725*	β2-MG	-0.215	0.039
93	*Janibacter A massiliensis*	RBP	-0.225	0.031
94	*Corynebacterium kefirresidentii*	RBP	-0.302	0.003
95	*Corynebacterium simulans*	RBP	-0.233	0.025
96	*Rhizorhabdus sp018006725*	RBP	-0.317	0.002
97	*Burkholderia sp018375725*	RBP	-0.214	0.041
98	*Ralstonia pseudosolanacearum*	RBP	-0.225	0.031
99	*Corynebacterium kefirresidentii*	DBIL	0.397	<0.001
100	*Corynebacterium simulans*	DBIL	0.314	0.002
101	*UBA7173 sp001689485*	DBIL	0.238	0.022
102	*Rhizorhabdus sp018006725*	DBIL	0.211	0.043
103	*Sphingobium yanoikuyae*	DBIL	0.220	0.035
104	*Ralstonia sp016429285*	DBIL	0.256	0.014
105	*Comamonas sp902175065*	DBIL	-0.284	0.006
106	*Nitrotoga sp021731185*	DBIL	-0.229	0.028
107	*Pseudomonas E alcaliphila B*	DBIL	0.263	0.011
108	*Bifidobacterium infantis*	IBIL	0.228	0.029
109	*Janibacter sp024362365*	IBIL	0.299	0.004
110	*Corynebacterium simulans*	IBIL	0.206	0.049

111	*Gordonia bronchialis*	IBIL	0.301	0.004
112	*Lawsonella clevelandensis A*	IBIL	0.299	0.004
113	*Campylobacter D coli*	IBIL	0.210	0.044
114	*Sphingobium yanoikuyae*	IBIL	0.276	0.008
115	*Burkholderia sp018375725*	IBIL	0.230	0.028
116	*Aquabacterium sp913773725*	IBIL	-0.333	0.001
117	*Achromobacter aegrifaciens*	IBIL	-0.228	0.029
118	*Achromobacter denitrificans*	IBIL	-0.229	0.028
119	*Achromobacter xylosoxidans*	IBIL	-0.223	0.032
120	*Bifidobacterium infantis*	TBIL	0.209	0.046
121	*Janibacter sp024362365*	TBIL	0.283	0.006
122	*Corynebacterium kefirresidentii*	TBIL	0.241	0.021
123	*Corynebacterium simulans*	TBIL	0.282	0.006
124	*Gordonia bronchialis*	TBIL	0.294	0.004
125	*Lawsonella clevelandensis A*	TBIL	0.323	0.002
126	*Cutibacterium avidum*	TBIL	0.209	0.046
127	*Campylobacter D coli*	TBIL	0.246	0.018
128	*Asticcacaulis excentricus A*	TBIL	-0.223	0.033
129	*Methylobacterium rhodesianum*	TBIL	0.252	0.015
130	*Mesorhizobium lusatiense*	TBIL	-0.227	0.030
131	*Hankyongella ginsenosidimutans*	TBIL	-0.224	0.032
132	*Sphingobium yanoikuyae*	TBIL	0.291	0.005
133	*Burkholderia sp018375725*	TBIL	0.265	0.011
134	*Ralstonia pseudosolanacearum*	TBIL	0.219	0.036
135	*Aquabacterium sp913773725*	TBIL	-0.267	0.010
136	*Achromobacter aegrifaciens*	TBIL	-0.273	0.009

137	*Achromobacter denitrificans*	TBIL	-0.281	0.007
138	*Achromobacter xylosoxidans*	TBIL	-0.228	0.029
139	*Alcaligenes phenolicus*	TBIL	-0.258	0.013
140	*Microbacterium lacticum*	NLR	-0.223	0.033
141	*Corynebacterium kefirresidentii*	NLR	-0.314	0.002
142	*Corynebacterium simulans*	NLR	-0.215	0.039
143	*Campylobacter D coli*	NLR	-0.249	0.017
144	*Elstera cyanobacteriorum*	NLR	0.212	0.043
145	*Afipia sp000497575*	NLR	-0.225	0.031
146	*Pseudomonas E sp019747075*	NLR	0.266	0.010
147	*Corynebacterium kefirresidentii*	HBG	0.270	0.009
148	*Corynebacterium sp000478175*	HBG	0.208	0.047
149	*Cutibacterium avidum*	HBG	0.233	0.026
150	*Cutibacterium granulosum*	HBG	0.308	0.003
151	*Clostridium neonatale*	HBG	0.209	0.045
152	*Bradyrhizobium mercantei*	HBG	-0.230	0.027
153	*Ralstonia sp001078575*	HBG	0.242	0.020
154	*Janibacter A massiliensis*	RBC	-0.209	0.045
155	*Corynebacterium sp000478175*	RBC	0.240	0.021
156	*Cutibacterium granulosum*	RBC	0.294	0.004
157	*Methylobacterium jeotgali*	RBC	0.208	0.047
158	*Janibacter A massiliensis*	HCT	-0.230	0.028
159	*Corynebacterium kefirresidentii*	HCT	0.256	0.014
160	*Corynebacterium sp000478175*	HCT	0.213	0.041
161	*Cutibacterium avidum*	HCT	0.226	0.031
162	*Cutibacterium granulosum*	HCT	0.300	0.004

163	*Clostridium neonatale*	HCT	0.235	0.024
164	*Agrobacterium tomkonis*	HCT	0.218	0.037
165	*Ralstonia sp001078575*	HCT	0.243	0.020
166	*Afipia sp000497575*	ALB	0.244	0.019
167	*Bradyrhizobium mercantei*	ALB	-0.219	0.036
168	*Ralstonia pseudosolanacearum*	ALB	0.213	0.042
169	*Acinetobacter johnsonii*	ALB	-0.216	0.038
170	*Pseudomonas E sp019747075*	ALB	-0.276	0.008
